# Decoy Nanozymes Enable Multitarget Blockade of Proinflammatory Cascades for the Treatment of Multi-Drug-Resistant Bacterial Sepsis

**DOI:** 10.34133/2022/9767643

**Published:** 2022-09-26

**Authors:** Xuancheng Du, Mingzhen Zhang, Huiting Zhou, Weijie Wang, Chengmei Zhang, Lei Zhang, Yuanyuan Qu, Weifeng Li, Xiangdong Liu, Mingwen Zhao, Kangsheng Tu, Yong-Qiang Li

**Affiliations:** ^1^Institute of Advanced Interdisciplinary Science, School of Physics, Shandong University, Jinan 250100, China; ^2^School of Basic Medical Sciences, Xi'an Key Laboratory of Immune Related Diseases, Xi'an Jiaotong University, Xi'an 710061, China; ^3^Institute of Pediatric Research, Children's Hospital of Soochow University, Suzhou 215025, China; ^4^Laboratory Animal Center of Shandong University, Jinan 250012, China; ^5^Department of Critical Care Medicine, the First Affiliated Hospital of Xi'an Jiaotong University, Xi'an 710061, China; ^6^Department of Hepatobiliary Surgery, the First Affiliated Hospital of Xi'an Jiaotong University, Xi'an 710061, China; ^7^Suzhou Research Institute, Shandong University, Suzhou 215123, China; ^8^College of Chemistry, Chemical Engineering and Materials Science, Shandong Provincial Key Laboratory of Clean Production of Fine Chemicals, Shandong Normal University, Jinan 250014, China

## Abstract

Sepsis is a life-threatening organ dysfunction characterized by severe systemic inflammatory response to infection. Effective treatment of bacterial sepsis remains a paramount clinical challenge, due to its astonishingly rapid progression and the prevalence of bacterial drug resistance. Here, we present a decoy nanozyme-enabled intervention strategy for multitarget blockade of proinflammatory cascades to treat multi-drug-resistant (MDR) bacterial sepsis. The decoy nanozymes (named MCeC@M*Φ*) consist mesoporous silica nanoparticle cores loaded with CeO_2_ nanocatalyst and Ce6 photosensitizer and biomimetic shells of macrophage membrane. By acting as macrophage decoys, MCeC@M*Φ* allow targeted photodynamic eradication of MDR bacteria and realize simultaneous endotoxin/proinflammatory cytokine neutralization. Meanwhile, MCeC@M*Φ* possess intriguing superoxide dismutase and catalase-like activities as well as hydroxyl radical antioxidant capacity and enable catalytic scavenging of multiple reactive oxygen species (ROS). These unique capabilities make MCeC@M*Φ* to collaboratively address the issues of bacterial infection, endotoxin/proinflammatory cytokine secretion, and ROS burst, fully cutting off the path of proinflammatory cascades to reverse the progression of bacterial sepsis. *In vivo* experiments demonstrate that MCeC@M*Φ* considerably attenuate systemic hyperinflammation and rapidly rescue organ damage within 1 day to confer higher survival rates (>75%) to mice with progressive MDR *Escherichia coli* bacteremia. The proposed decoy nanozyme-enabled multitarget collaborative intervention strategy offers a powerful modality for bacterial sepsis management and opens up possibilities for the treatment of cytokine storm in the COVID-19 pandemic and immune-mediated inflammation diseases.

## 1. Introduction

Sepsis is a life-threatening organ dysfunction caused by dysregulated host immune response to infection, which involves an initial overwhelming proinflammatory stage and subsequent immunosuppressive phase [1, 2]. Sepsis is reported to affect more than 49 million people every year and account for nearly 20% of all death globally and has been recognized as a worldwide health priority by the World Health Organization (WHO) [3, 4]. Bacteria are the primary pathogens of sepsis, and current treatments extensively used in clinical practice include empirical administration of broad-spectrum antibiotics, fluid resuscitation, and organ support [5, 6]. These approaches can support end-organ functions and may help manage bacterial infection. However, they have a minor sepsis therapeutic effect especially in the context of the prevalence of bacterial drug resistance [7, 8]. In addition to the standard care of sepsis, dozens of clinical trials of immunomodulators concerning various aspects of sepsis conditions have been undertaken in recent years [9, 10]. Despite conspicuous advantages and progress of these well-designed immunomodulators, limited success has been met as evidenced by persistent sepsis mortality [11]. These dilemmas in sepsis management desperately call for the innovation of medical intervention strategy.

Pathologically, the initial proinflammatory stage of sepsis provides a relatively feasible intervention window, based on the helpless clinical fact that once sepsis enters the immunosuppressive phase, its progression is very tricky to reverse accompanied by a sharp increase in patient mortality [12]. The proinflammatory stage of sepsis is initiated by the massive invasion of pathogens, which introduce pathogen-associated molecular patterns (PAMPs) such as endotoxins into the host system [13]. The PAMPs can then be recognized by Toll-like receptors expressed on the extracellular surface of immune cells to activate host innate immunity, triggering the secretion of proinflammatory cytokines such as tumor necrosis factor *α* (TNF-*α*), interleukin-6 (IL-6), and interleukin-1*β* (IL-1*β*) [14]. Subsequently, these proinflammatory cytokines continuously recruit more inflammatory immune cells to the persistent infection sites, where as daunting “root” elicit excessive immune system activation and fuel systemic inflammation [15, 16]. Concomitantly, immoderate production of proinflammatory cytokines and the burst of reactive oxygen species (ROS) occurred, resulting in cytokine storm and inevitably causing tissue damage and multiple organ dysfunction [17, 18]. Researchers have attempted to intervene sepsis by targeting specific proinflammatory mediators, such as counteracting the burst of ROS with nanozymes [19–21]. Many nanozymes have been found to exhibit excellent antioxidant capacity, either by themselves or after surface modification [22–25]. They have shown significant ROS scavenging capacity in the treatment of proinflammatory sepsis [26, 27]. In addition to this, photodynamic eradication of multi-drug-resistant (MDR) bacteria is also commonly used as a therapeutic option [28, 29]. However, previous clinical trials have demonstrated that intervention means individually targeting certain proinflammatory mediators cannot inhibit the progression of the proinflammatory stage of sepsis in time to reduce the mortality rate [30, 31]. Therefore, from the perspective of the full path of the occurrence and progression of the proinflammatory stage, we hypothesize that multitarget (pathogens, PAMPs, proinflammatory cytokines, and ROS) collaborative intervention may be more effective in tackling sepsis. However, an integrated intervention system with combined capabilities of pathogen eradication, PAMP neutralization, proinflammatory cytokine sequestration, and ROS scavenging remains elusive.

**Figure 1:**
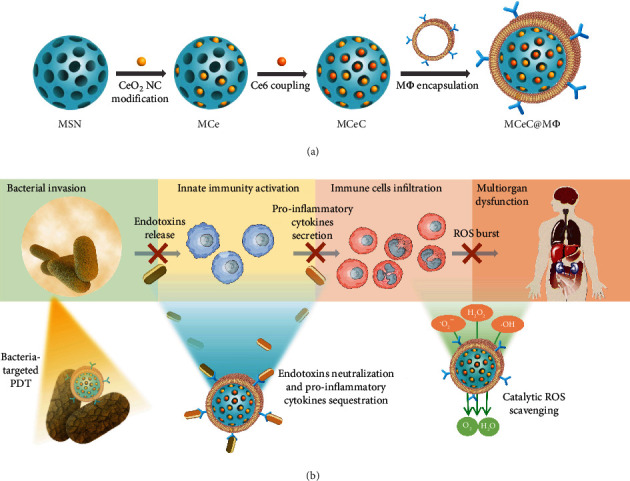
Decoy nanozyme-enabled treatment of MDR bacterial sepsis. (a) Schematic illustration of MCeC@M*Φ* preparation. (b) Conceptual illustration of multitarget blockade of proinflammatory cascades in MDR bacterial sepsis based on MCeC@M*Φ*.

Herein, we present a multitarget collaborative intervention strategy for rescuing overwhelming inflammation to treat multi-drug-resistant (MDR) bacterial sepsis based on decoy nanozymes. The decoy nanozymes (termed MCeC@M*Φ*) are fabricated by incorporating mesoporous silica nanoparticles (MSN) with cerium oxide nanocatalyst (CeO_2_ NC) and photosensitizer of chlorin e6 (Ce6) and then encapsulating it with macrophage membranes (M*Φ*) (as depicted in Figure 1(a)). The MCeC@M*Φ* possess an antigenic exterior the same as macrophages due to M*Φ* camouflage and inherit a remarkable ability to bind endotoxins and proinflammatory cytokines. By acting as macrophage decoys, MCeC@M*Φ* allow simultaneous endotoxin neutralization and proinflammatory cytokine sequestration, protecting host immune cells from overactivation and cutting off the chain of inflammatory actions. Meanwhile, the loaded CeO_2_ NC bestows the MCeC@M*Φ* with intriguing superoxide dismutase- (SOD-) and catalase- (CAT-) like activities as well as hydroxyl radical antioxidant capacity (HORAC), enabling rapid scavenging of multiple ROS to relieve systemic oxidative stress and mitigate tissue damage. More importantly, intrinsic bacteria targeting of M*Φ* and the coexistence of Ce6 photosensitizer and CeO_2_ NC scavenger empower MCeC@M*Φ* to realize targeted photodynamic therapy (PDT) of MDR bacteria without PDT-aggravated inflammation, digging out the “root” of sepsis inflammatory fluxes. By collaboratively addressing the issues of bacterial infection, endotoxin and proinflammatory cytokine secretion, and ROS burst to block proinflammatory cascades, the MCeC@M*Φ*-based “weeding and uprooting” intervention strategy may offer a powerful paradigm for clinical bacterial sepsis management (Figure 1(b)).

## 2. Results

### 2.1. Preparation and Characterization of Decoy Nanozymes

**Figure 2:**
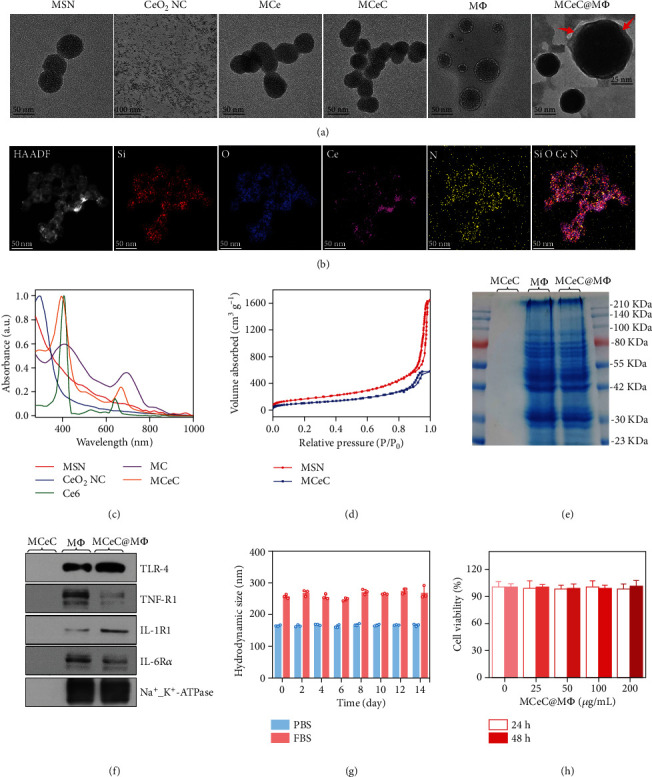
Characterization of decoy nanozymes. (a) Representative TEM images of MSN, CeO_2_ NC, MCe, MCeC, M*Φ*, and MCeC@M*Φ*. (b) High-angle annular dark-field scanning TEM image and corresponding EDX mapping of MCeC@M*Φ*. (c) UV-vis absorption spectra of MSN, CeO_2_ NC, Ce6, MC, and MCeC. (d) N_2_ absorption/desorption isotherms of MSN and MCeC. (e) SDS-PAGE protein profiles of MCeC, M*Φ*, and MCeC@M*Φ*. (f) Western blotting analysis for typical protein markers of TLR-4, TNF-R1, IL-1R1, and IL-6R*α* in MCeC, M*Φ*, and MCeC@M*Φ*. (g) Hydrodynamic diameters of MCeC@M*Φ* in PBS buffer (0.01 M, pH 7.4) and fetal bovine serum (FBS) during two weeks of storage. (h) Viability of the human umbilical endothelial cells (HUVEC) after incubation with MCeC@M*Φ* at various concentrations of Ce element for 24 and 48 h, respectively. In (g) and (h), the values of hydrodynamic diameter and cell viability represent the mean of the three independent experiments, and the error bars indicate the standard deviation (SD) from the mean.

The decoy nanozymes of MCeC@M*Φ* were prepared by consecutively decorating MSN (size around 65 nm) with CeO_2_ NC (size around 4 nm) and Ce6 molecule through a nucleophilic substitution reaction and chemical covalent coupling method [32, 33], respectively, and then encapsulating it with M*Φ* (Figures 1(a) and 2(a)). The conjugation of CeO_2_ NC to MSN was confirmed by the X-ray powder diffraction (XRD) pattern (Figure S1) and Fourier transform infrared (FTIR) spectrum (Figure S2) of CeO_2_ NC-decorated MSN (MCe), as well as energy-dispersive X-ray (EDX) elemental mapping of MCeC@M*Φ* in which Si and Ce elements (the ratio of Si and Ce was around 15) were clearly observed (Figure 2(b) and Figure S3). UV-vis absorption spectra showed that Ce6-decorated MCe (MCeC) and Ce6-decorated MSN (MC) exhibited the characteristic absorption peaks of Ce6 at around 400 and 650 nm [34], indicating the successful linkage of Ce6 (Figure 2(c) and Figure S4). The covalent attachment of Ce6 could be confirmed by the negligible Ce6 release in MCeC during one-day storage (Figure S5). The amount of Ce6 attached in MCeC was determined to be 10 *μ*g/mL by calculating the concentration of free Ce6 in the reaction solution before and after covalent coupling based on the standard absorption curve of Ce6 (Figure S6). N_2_ adsorption-desorption experiments showed that the Brunauer-Emmett-Teller (BET) surface area and median pore volume of MSN became significantly decreased after CeO_2_ NC and Ce6 decoration (Figure 2(d) and Figure S7). From the transmission electron microscopy (TEM) images of the decoy nanozymes, the MCeC@M*Φ* were found to possess regular and well-defined core-shell structures with the average size of 71.2 ± 1.9 nm, in which the MCeC cores were wrapped with thin shells (red arrows), revealing the encapsulation of M*Φ* (Figure 2(a)). In addition, the whole preparation process of MCeC@M*Φ* could also be monitored and confirmed by the reversed zeta potential and increased hydrodynamic size results (Figure S8 and S9).

Through M*Φ* coating, the MCeC@M*Φ* are expected to inherit the same antigenic exterior of macrophages. To demonstrate this, sodium dodecyl sulfate-polyacrylamide gel electrophoresis (SDS-PAGE) was performed to analyze the protein composition profile of MCeC@M*Φ*. As shown in Figure 2(e), the overall protein profile of MCeC@M*Φ* was nearly identical to that of purified M*Φ*, while no protein signal was detected in MCeC, indicating the effective translocation of M*Φ* proteins onto the MCeC. Furthermore, the expression of typical cell membrane receptors including Toll-like receptor 4 (TLR-4), tumor necrosis factor receptor 1 (TNF-R1), interleukin-6 receptor *α* (IL-6R*α*), and interleukin-1 receptor 1 (IL-1R1) [35–37] was clearly identified in MCeC@M*Φ* based on western blot analysis (Figure 2(f)). Moreover, the prepared MCeC@M*Φ* were demonstrated to exhibit long-term size stability and outstanding in vitro biocompatibility based on the results of dynamic light scattering measurement and cell antiproliferation assay (Figures 2(g) and 2(h) and Figure S10). This excellent size stability and biocompatibility might be ascribed to the stabilizing effect of hydrophilic surface glycans on M*Φ* [38].

### 2.2. In Vitro Endotoxin Neutralization and Proinflammatory Cytokine Sequestration

The robust expression of cell membrane receptors (TLR-4, TNF-R1, IL-1R1, and IL-6R*α*) in MCeC@M*Φ* could empower the decoy nanozymes to bind endotoxins and proinflammatory cytokines [39], enabling endotoxin neutralization and proinflammatory cytokine sequestration. To demonstrate this, the separation of typical endotoxin and proinflammatory cytokine suspensions was carried out. Here, two kinds of decoy nanozymes that fuse with cell membranes derived from normal macrophages (MCeC@n-M*Φ*) and LPS-stimulated macrophages (MCeC@s-M*Φ*), respectively, were employed (Figure S11). The endotoxin- and proinflammatory cytokine-binding ability of decoy nanozymes was first assessed. As shown in Figure S12 and S13, the MCeC@n-M*Φ* and MCeC@s-M*Φ* possessed the superior binding ability for the selected endotoxin of lipopolysaccharide (LPS) and proinflammatory cytokines (TNF-*α*, IL-6, and IL-1*β*) compared to control group (PBS and MCeC). The difference in the binding amount of decoy nanozymes toward LPS (ng level) and proinflammatory cytokines of TNF-*α*, IL-6, and IL-1*β* (pg level) is closely related to the expression of receptors of TLR-4, TNF-R1, IL-6R*α*, and IL-1R1 in the membrane of macrophages (Figure 2(f)). Subsequently, the performance of decoy nanozymes for endotoxin neutralization and proinflammatory cytokine sequestration was evaluated. As shown in Figure S14, the decoy nanozymes exhibited an obvious concentration-dependent neutralization/sequestration capability for endotoxin and proinflammatory cytokines, and remarkable neutralization/sequestration rates were achieved for the mixed solution of LPS, TNF-*α*, IL-6, and IL-1*β*. In addition, MCeC@s-M*Φ* were found to obtain relatively higher neutralization/sequestration rates than MCeC@n-M*Φ*, which is consistent with the fact of enhanced expression of cell membrane receptors in LPS-stimulated macrophages.

**Figure 3:**
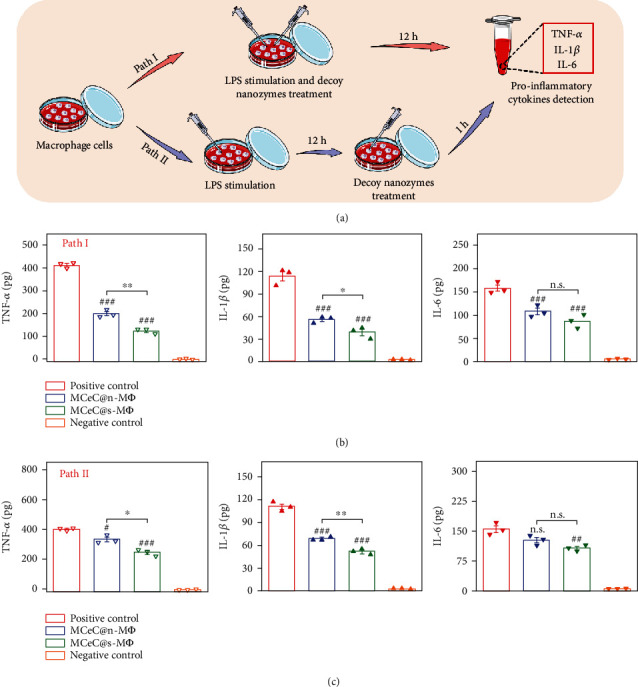
*In vitro* performance of decoy nanozymes for endotoxin neutralization and proinflammatory cytokine sequestration. (a) Schematic illustration of experimental paths (path I and path II) employed to evaluate the inhibitory performance of decoy nanozymes on the secretion of proinflammatory cytokines by J774 macrophage cells upon LPS stimulation. (b, c) The secretion of proinflammatory cytokines (TNF-*α*, IL-1*β*, and IL-6) by J774 macrophage cells treated by two decoy nanozymes (MCeC@n-M*Φ* and MCeC@s-M*Φ*) in the path I and path II. The treatment group of PBS was used as the positive control, while the normal J774 macrophage cell without LPS stimulation was used the negative control. In (b) and (c), the values of proinflammatory cytokines amount secreted represent the mean of the three independent experiments, and the error bars indicate the SD from the mean. # indicates the contrasts between experimental groups and positive control. ^#/^^∗^*P* < 0.05, ^##/^^∗∗^*P* < 0.01, ^###^*P* < 0.001, and ^n.s.^*P* > 0.05.

Theoretically, the robust endotoxin neutralization and proinflammatory cytokine sequestration capability of decoy nanozymes can protect host immune cells from overactivation and ameliorate their inflammatory states. To demonstrate this, the secretion of proinflammatory cytokines (TNF-*α*, IL-6, and IL-1*β*) by J774 macrophage cells upon LPS stimulation in the absence/presence of decoy nanozymes was investigated. Here, two different experimental paths were carried out to better simulate the inflammatory conditions of macrophages in bacterial sepsis. Specifically, J774 macrophage cells were coincubated with LPS and decoy nanozymes in the path I to simulate the pathological response of early sepsis, while J774 macrophage cells were first stimulated by LPS for 12 h followed by decoy nanozyme treatment in the path II to evaluate their performance in progressive sepsis (Figure 3(a)). As shown in Figures 3(b) and 3(c), the secretion of proinflammatory cytokines of TNF-*α*, IL-6, and IL-1*β* was both significantly inhibited in the two experimental conditions (path I and II) by decoy nanozymes (MCeC@n-M*Φ* and MCeC@s-M*Φ*), indicating the mitigation of inflammatory states in LPS-stimulated J774 macrophage cells after decoy nanozyme treatment. In addition to the inhibition of proinflammatory cytokine secretion, decoy nanozymes were found to enhance the secretion of anti-inflammatory cytokine of interleukin-10 (IL-10) and the expression of Arg-1 (M2 macrophage marker) in LPS-stimulated J774 macrophage cells, showing a positive effect on the polarization of macrophage phenotype from M1 to M2 (Figure S15 and S16) [40]. Compared to MCeC@n-M*Φ*, MCeC@s-M*Φ* exhibited relatively better performance for the inhibition of proinflammatory cytokine secretion as well as the enhancement of macrophage M1/M2 polarization (Figures 3(b) and 3(c) and Figure S16) and was chosen in the following experiments.

### 2.3. In Vitro ROS Scavenging

**Figure 4:**
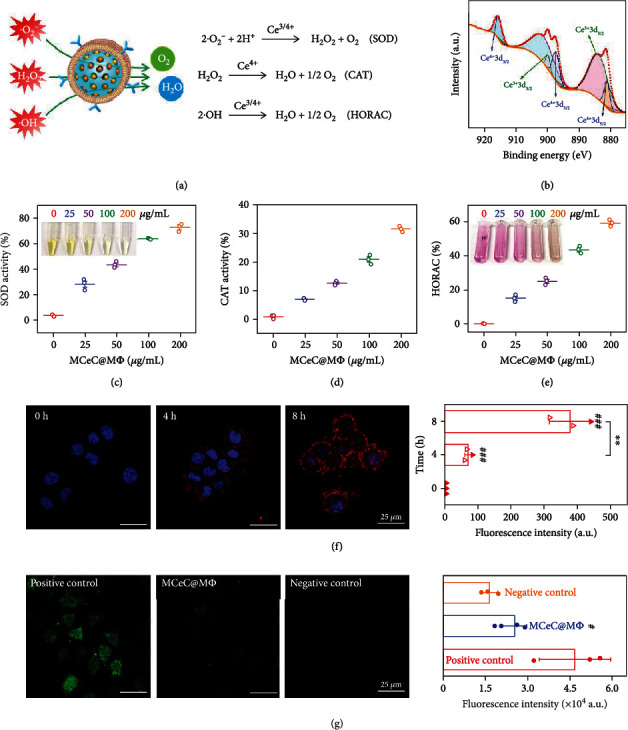
*In vitro* performance of decoy nanozymes for ROS scavenging. (a) Schematic illustration of the SOD, CAT, and HORAC activity of MCeC@M*Φ* for multiple ROS scavenging. (b) XPS spectrum of MCeC@M*Φ*. (c) SOD-like activity of MCeC@M*Φ* with different concentrations. The inset shows the corresponding photographs of SOD test solution in the presence of different concentrations of MCeC@M*Φ*. (d) CAT-like activity of MCeC@M*Φ* with different concentrations. (e) HORAC performance of MCeC@M*Φ* with different concentrations. The inset shows the corresponding photographs of HORAC test solution in the presence of different concentrations of MCeC@M*Φ*. (f) Representative overlapping CLSM images and corresponding fluorescence intensities of J774 macrophage cells incubated with MCeC@M*Φ* at various time points. The group of 0 h was used as the positive control. (g) Representative DCF staining images and corresponding fluorescence intensities of J774 macrophage cells with ROS hyperactivity state upon MCeC@M*Φ* treatment. The treatment group of PBS was used as the positive control, while the normal J774 macrophage cell without ROS hyperactivity state was used the negative control. In (f) and (g), the values of fluorescence intensity represent the mean of the three independent experiments, and the error bars indicate the SD from the mean. # indicates the contrasts between experimental groups and positive control. ^#^*P* < 0.05, ^∗∗^*P* < 0.01, and ^###^*P* < 0.001.

As an effective antioxidant, CeO_2_ NC possesses intrinsic SOD- and CAT-like activities as well as HORAC due to the shift between Ce^3+^ and Ce^4+^ valence state [41, 42]. In principle, MCeC@M*Φ* will inherit these unique enzymatic properties of CeO_2_ NC and can catalyze the conversion of superoxide anion (∙O_2_^–^), hydrogen peroxide (H_2_O_2_), and hydroxyl radical (∙OH) into water and oxygen, enabling effective scavenging of multiple ROS (Figure 4(a)). X-ray photoelectron spectroscopy (XPS) analysis identified the mixed Ce^3+^/Ce^4+^ valence state in MCeC@M*Φ*, heralding the potential multienzyme characteristics of decoy nanozymes (Figure 4(b) and Figure S17). To demonstrate this, the SOD- and CAT-like activities as well as HORAC performance of MCeC@M*Φ* were systematically evaluated. As shown in Figure 4(c), MCeC@M*Φ* inhibited the formazan (yellow color with an absorbance around 450 nm) generation reaction between ∙O_2_^–^ and WST-1 tetrazolium dye due to the scavenging ∙O_2_^–^ and showed encouraging SOD-like activity (around 80% under 200 *μ*g/mL) comparable to CeO_2_ NC. In addition, MCeC@M*Φ* were proved to catalyze the decomposition of H_2_O_2_ to produce oxygen and exhibited obvious CAT-like activity (around 35%) under the concentration of 200 *μ*g/mL (Figure 4(d) and Figure S18). Furthermore, ∙OH was found to be efficiently decomposed in the presence of MCeC@M*Φ* based on the color reaction between ∙OH and Griess agent, possessing robust HORAC activity (around 60%) under the concentration of 200 *μ*g/mL (Figure 4(e)).

Subsequently, MCeC@M*Φ* were incubated with J774 macrophage cells, and their cell uptake ability and the performance for intracellular ROS scavenging were evaluated. Figure 4(f) shows the confocal laser fluorescence microscopy (CLSM) images of J774 macrophage cells incubated with MCeC@M*Φ* at various time points. The fluorescence of MCeC@M*Φ* (coming from loaded Ce6) in J774 macrophage cells was clearly observed and continued to increase with the extension of incubation time, demonstrating wonderful macrophage uptake efficiency and paving the way for intracellular ROS scavenging. To perform intracellular ROS scavenging experiment, J774 macrophage cells with ROS hyperactivity state were treated by MCeC@M*Φ*, and dichlorofluorescein (DCF, a fluorescent marker generated from dichlorodihydrofluorescein dye by ROS) staining was carried out [43]. As shown in Figure 4(g), J774 macrophage cells in the treatment group of MCeC@M*Φ* showed much lower DCF fluorescence than the positive control (PBS), and their fluorescence intensities were further found to be indistinguishable from that of J774 cells without ROS hyperactivity state (negative control), indicating that the excessive intracellular ROS was effectively eliminated by MCeC@M*Φ*.

### 2.4. In Vitro MDR Bacterial Elimination

**Figure 5:**
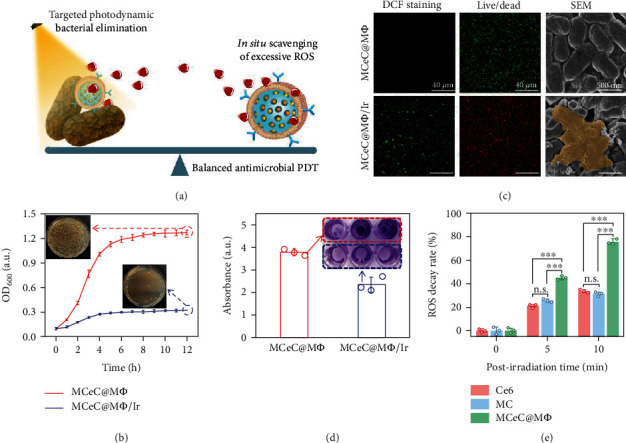
*In vitro* performance of decoy nanozymes for MDR bacteria elimination. (a) Schematic illustration of MCeC@M*Φ*-based balanced antimicrobial PDT paradigm enabling targeted photodynamic bacterial elimination and excessive ROS scavenging. (b) Growth curves of MDR *E. coli* treated by MCeC@M*Φ* and MCeC@M*Φ*/Ir, respectively, and the inset shows the corresponding photographs of culture plates of MDR *E. coli* taken from the two treatment groups at the time point of 12 h. (c) Representative DCF staining, live/dead, and SEM images of MDR *E. coli* in the treatment groups of MCeC@M*Φ* and MCeC@M*Φ*/Ir, respectively. (d) Crystal violet staining image and its corresponding absorbance for mature MDR *E. coli* biofilm treated by MCeC@M*Φ* and MCeC@M*Φ*/Ir, respectively. (e) ROS decay rate in MCeC@M*Φ*, Ce6, and MC within 10 min after laser irradiation (660 nm laser, 0.8 W/cm^2^, 5 min), respectively. In (b), (d), and (e), the values of OD_600_, crystal violet absorbance, and ROS decay rate represent the mean of the three independent experiments, and the error bars indicate the SD from the mean. ^∗∗∗^*P* < 0.001 and ^n.s.^*P* > 0.05.

By integrating bacteria-targeting component (M*Φ*), photosensitizer (Ce6), and ROS scavenger (CeO_2_ NC), MCeC@M*Φ* are expected to enable targeted photodynamic eradiation of bacteria on one hand and scavenge excessive ROS to deracinate PDT-aggravated inflammation on the other hand (Figure 5(a)). To verify this expectation, the antimicrobial PDT performance of MCeC@M*Φ* was first evaluated. As shown in Figure 5(b), the growth of Gram-negative MDR bacterial strain of *Escherichia coli* (*E. coli*) was extremely inhibited in the treatment group of MCeC@M*Φ* plus laser irradiation (MCeC@M*Φ*/Ir) compared to MCeC@M*Φ*, showing excellent antimicrobial PDT activity. This antimicrobial result was consistent with the phenomenon of higher levels of intracellular ROS in MDR *E. coli* as evidenced by DCF staining assay, indicating ROS-medicated photodynamic bacterial eradication (Figure 5(c)). To further investigate the antimicrobial mechanism behind, live/dead bacterial staining assay and scanning electronic microscopy- (SEM-) based bacterial morphology study were performed. As shown in Figure 5(c), MDR *E. coli* showed smooth bodies and was stained green by SYTO 9 dye in the treatment group of MCeC@M*Φ*, exhibiting normal survival state. In sharp contrast, cellular deformation and surface collapse as well as propidium iodide dye (red color, only penetrate microbes with destroyed structure) staining were clearly observed in MDR *E. coli* treated by MCeC@M*Φ*/Ir, suggesting a cell wall and membrane disruption-involved bactericidal mechanism. In addition to planktonic bacteria, the PDT performance of MCeC@M*Φ* toward biofilm (the predominant form of bacteria *in vivo*) was also assessed. Crystal violet staining assay demonstrated that MCeC@M*Φ* not only inhibited the formation of MDR *E. coli* biofilm but also effectively destroyed mature MDR *E. coli* biofilm under laser irradiation, exhibiting robust biofilm eradication capability (Figure 5(d) and Figure S19).

The residual ROS after photodynamic bacterial elimination is a nonnegligible risk issue, which inevitably cases damage to surrounding tissue and constitutes the main bottleneck of conventional PDT [44]. The coexistence of Ce6 photosensitizer and CeO_2_ NC ROS scavenger in MCeC@M*Φ* offers a promising way to break this bottleneck by deracinating PDT-aggravated inflammation. To demonstrate the feasibility of MCeC@M*Φ* for the elimination of PDT-aggravated inflammation, the decay of ROS in MCeC@M*Φ* after irradiation was investigated using singlet oxygen sensor green (SOSG) [45]. As shown in Figure S20, ROS generated in the photodynamic process was continuously attenuated over time, and enhanced ROS decay was found in MCeC@M*Φ* compared to that of Ce6 and MC after irradiation, respectively, indicating the critical role of CeO_2_ NC scavenger. Quantitatively, around 75% of ROS was scavenged at 10 min postirradiation in MCeC@M*Φ*, while the ROS decay rate in Ce6 and MC at 10 min postirradiation was just 32% and 30%, respectively, showing the outstanding ability for the elimination of PDT-aggravated inflammation (Figure 5(e)). The excellent bactericidal performance and PDT-aggravated inflammation deracination talent of MCeC@M*Φ* provide a balanced antimicrobial PDT paradigm, laying a solid foundation for subsequent in vivo bacterial elimination.

### 2.5. In Vivo MDR Bacterial Sepsis Treatment

**Figure 6:**
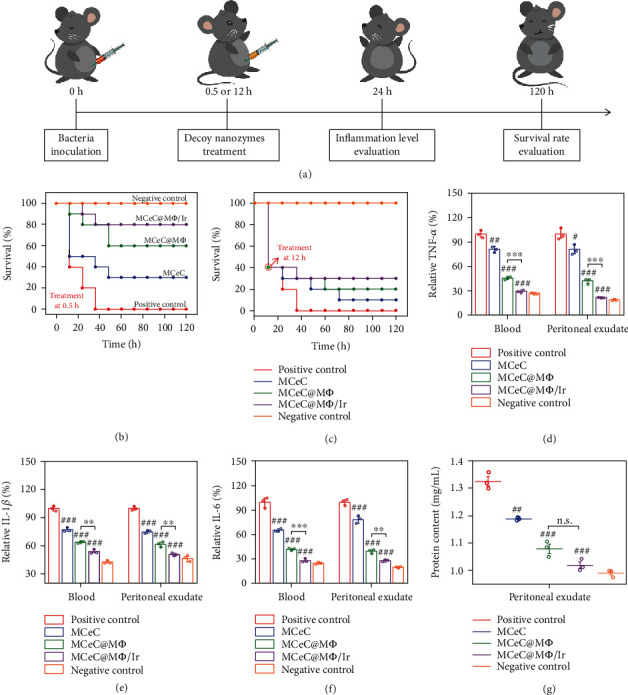
*In vivo* performance of decoy nanozymes for MDR bacterial sepsis treatment. (a) Experimental timeline and schematic representation of decoy nanozymes for *in vivo* treatment of MDR bacterial sepsis. (b) Survival rate of septic mice treated 0.5 h after bacterial inoculation in different experimental groups. (c) Survival rate of septic mice treated 12 h after bacterial inoculation in different experimental groups. (d–f) Proinflammatory cytokines of TNF-*α*, IL-1*β*, and IL-6 in the blood and peritoneal exudate of septic mice treated 0.5 h after bacterial inoculation in different experimental groups. (g) Protein content in the peritoneal exudate of septic mice treated 0.5 h after bacterial inoculation in different experimental groups. The treatment group of PBS was used as the positive control, while the group of normal healthy mice was used as the negative control. In (d), (e), (f), and (g), the values of relative TNF-*α*, IL-1*β*, and IL-6 as well as protein content represent the mean of the three independent experiments, and the error bars indicate the SD from the mean. # indicates the contrasts between experimental groups and positive control. ^#^*P* < 0.05, ^##/^^∗∗^*P* < 0.01, ^###/^^∗∗∗^*P* < 0.001, and ^n.s.^*P* > 0.05.

The multiple abilities (endotoxin neutralization and proinflammatory cytokine sequestration, ROS scavenging, and bacterial elimination) of decoy nanozymes make them an ideal candidate for *in vivo* bacterial sepsis treatment via collaboratively blocking overwhelming inflammation cascades. To verify the therapeutic effect of decoy nanozymes, a mouse model of MDR bacterial sepsis was first established by inoculating lethal doses of 2 × 10^8^ colony-forming units (CFU) of MDR *E. coli* through intraperitoneal (*i.p.*) injection to trigger an aberrant inflammatory response. Septic mice were then treated by intraperitoneally injecting MCeC@M*Φ* at two time points (0.5 and 12 h) after bacterial inoculation to evaluate their therapeutic performance in different pathological states (early and progressive period) of sepsis (Figure 6(a)). Four treatment groups were divided including PBS (positive control), MCeC, MCeC@M*Φ*, and MCeC@M*Φ*/Ir, and the group of normal healthy mice was used as the negative control. Figure 6(b) shows the survival profiles of septic mice treated 0.5 h after bacterial inoculation (early sepsis). Compared with the positive control (PBS) with a 100% mortality rate, the survival was greatly enhanced in the other three treatment groups, and an extremely high survival rate (80%) was obtained in the treatment group of MCeC@M*Φ*/Ir. Figure 6(c) shows the survival profiles of septic mice treated 12 h after bacterial inoculation (progressive sepsis). It was found that the septic mice already had a lot of casualties (only 40% of survivors) at the time point of treatment, indicating an extremely dangerous stage of rapid sepsis progression. Remarkably, most of the survivors (75%) were rescued under the treatment of MCeC@M*Φ*/Ir, showing outstanding performance for *in vivo* sepsis intervention and management.

To ascertain the mechanism behind the wonderful result of survival rate, the inflammation level of septic mice treated 0.5 h after bacterial inoculation in the four treatment groups was first quantitatively assessed. As shown in Figures 6(d)–6(f), the amount of typical proinflammatory factors (TNF-*α*, IL-6, and IL-1*β*) in blood and peritoneal exudate of septic mice under the treatment of MCeC@M*Φ*/Ir was efficiently reduced to near-normal levels (negative control) on the 1^st^ day of treatment. Apart from proinflammatory factor measurement, protein permeability in peritoneal exudate that represents vasculature integrity and is associated with inflammation was also evaluated [46]. As shown in Figure 6(g), a much lower protein concentration in the peritoneal exudate of septic mice was found in the treatment group of MCeC@M*Φ*/Ir, indicating extremely weak protein permeability. The crossvalidating proinflammatory factor and protein permeability results demonstrated that the hyperinflammation of septic mice was rapidly and effectively ameliorated upon decoy nanozyme treatment. In addition, MDR *E. coli* bacteria collected from blood, peritoneal exudate, and major organs (kidney, spleen, and liver) of septic mice treated 0.5 h after bacterial inoculation were counted on the 1^st^ day of treatment to evaluate the actual antimicrobial efficacy. It was found that the number of bacterial colony was positively correlated with the survival of septic mice, and bacterial burden was remarkably reduced in the treatment group of MCeC@M*Φ*/Ir (Figure S21). Alleviated proinflammatory factor secretion and reduced bacterial burden in turn circumvent organ damage of septic mice. Figure S22 and Figure S23 show the histological analysis (H&E staining) of organs of septic mice treated 0.5 h after bacterial inoculation under different treatment conditions. It was found that the damage of the liver and kidney of septic mice featuring severe inflammatory cell infiltration in perivascular area and spotty hepatocellular necrosis accompanied by lymphocytic infiltration was tremendously alleviated in the treatment group of MCeC@M*Φ*/Ir. These multidimensional experimental results described above strongly prove the feasibility of decoy nanozymes for *in vivo* treatment of MDR bacterial sepsis.

### 2.6. Biocompatibility Investigation of Decoy Nanozymes

The potential biotoxicity of nanomaterials is a key obstacle to its clinical transformation [47]. By considering the good biocompatibility of the components of MSN, CeO_2_ NC, and Ce6 as well as the biomimetic coating of M*Φ*, the decoy nanozymes of MCeC@M*Φ* have predictable outstanding biosafety in vitro and in vivo. The human umbilical vein endothelial cells (HUVEC) incubated with MCeC@M*Φ* were found to possess high viability based on the MTT result shown in Figure 2(h) and Figure S10, confirming their wonderful biocompatibility *in vitro*. To assess the biosafety effect of decoy nanozymes *in vivo*, healthy mice were intraperitoneally injected with MCeC@M*Φ*, and the tissue distribution of Ce element and blood biochemical assay as well as organ histopathological analysis were performed. As shown in Figure S24, a relatively higher amount of Ce element was observed in the liver and spleen, indicating possible liver- and spleen-based metabolic pathway of decoy nanozymes. Figure S25 and S26 show the blood routine and blood biochemical results of healthy mice on the 5^th^ day of decoy nanozyme postinjection. It was found that there was no obvious difference in blood biochemical indicators detected between the mice injected with MCeC@M*Φ*, MCeC@M*Φ*/Ir, and PBS (control), indicating the negligible damage of decoy nanozymes to the metabolism of the liver and kidney of mice. In addition, no lesions and inflammation were found in the main organs of the mice injected with MCeC@M*Φ* compared to the control from the histopathological staining images, exhibiting outstanding *in vivo* biocompatibility (Figure S27). This confirmed that excellent *in vitro* and *in vivo* biocompatibility of MCeC@M*Φ* decoy nanozymes lays a solid foundation for its future clinical transformation.

## 3. Discussion

From the perspective of cutting off the full path of the occurrence and progression of the proinflammatory phase induced jointly by pathogens, PAMPs, proinflammatory cytokines, and ROS, we propose a multitarget collaborative intervention strategy for rescuing bacterial sepsis based on decoy nanozymes. The decoy nanozymes are fabricated by wrapping CeO_2_ nanocatalyst and Ce6 photosensitizer-loaded MSN cores with cell membranes derived from macrophages. Regarding the culprit of the pathogen, the decoy nanozymes allow targeted photodynamic eradication of bacteria without PDT-aggravated inflammation, digging out the “root” of sepsis inflammatory fluxes. In addition, by acting as macrophage decoys, the decoy nanozymes realize simultaneous endotoxin neutralization and proinflammatory cytokine sequestration, protecting host immune cells from overactivation. Meanwhile, the decoy nanozymes enable rapid scavenging of multiple ROS via enzymatic reaction, relieving systemic oxidative stress and mitigating tissue damage. By collaboratively addressing the virulence factors of bacteria, endotoxins, proinflammatory cytokines, and ROS, the decoy nanozymes rapidly attenuate and reverse *in vivo* systemic hyperinflammation and ultimately confer considerably higher survival rates (>75%) to mice with progressive MDR *E. coli* bacteremia. The decoy nanozyme-based multitarget collaborative intervention strategy offers a powerful modality for rescuing overwhelming inflammation in MDR bacterial sepsis, potentially shifting the current paradigm of sepsis management. Predictably, the fascinating decoy nanozymes also open up possibilities for tackling overwhelming inflammation in the COVID-19 pandemic and severe immune-mediated inflammation diseases [48, 49].

## 4. Materials and Methods

### 4.1. Materials

Cetyltrimethylammonium chloride (CTAC), triethanolamine (TEA), tetraethyl orthosilicate (TEOS), mesitylene, 3-aminopropyl triethoxysilane (APTES), cerium acetate, oleylamine, xylene, citric acid, 2-bromo-2-methylpropionic acid (BMPA), chlorin e6 (Ce6), ethylenediaminetetraacetic acid (EDTA), N-(3-(dimethylamino)propyl-N′-ethylcarbodiimide) hydrochloride (EDC), N-hydroxysulfosuccinimide sodium salt (NHS), cell counting kit-8 (CCK-8), and 2,7-dichlorodifluorfluorescein diacetate (DCFH-DA) were obtained from Sigma-Aldrich. SOD test kit, CAT test kit, and HORAC test kit were purchased from Nanjing Jiancheng Bioengineering Research Institute. ELISA kits of TNF-*α*, IL-1*β*, IL-6, IL-10, and LPS were purchased from Beijing Boning Biotechnology Co., Ltd. Live/dead bacterial staining agent and singlet oxygen sensor green (SOSG) were purchased from Thermo Fisher. All other chemicals were obtained from Adamas-beta and used without further purification. Deionized (DI) water (Millipore Milli-Q grade, 18.2 M*Ω*) was used in all the experiments.

### 4.2. Preparation of Decoy Nanozymes

MSN was synthesized according to the previously reported method [32]. In brief, 2 g of CTAC and 0.02 g of TEA were dissolved in 20 mL of deionized water and stirred vigorously at 95°C for 1 h. Then, 1.5 mL of mesitylene and 1.5 ml of TEOS were added to the mixed solution. After 1 h reaction, the MSN precipitate was collected, repeatedly cleaned by the mixed solution of hydrochloric acid and ethanol to remove CTAC, and finally dispersed in ethanol. 200 *μ*L of APTES was subsequently added into the obtained MSN solution and refluxed at 65°C for 4 h to prepare aminated MSN. CeO_2_ NC was synthesized according to the previously reported method [50]. Briefly, 0.43 g of cerium acetate and 3.25 g of oleylamine were dissolved in 15 mL of xylene and reacted overnight at room temperature. Then, the solution was heated to 90°C in argon atmosphere, and 1 mL of deionized water was quickly injected and aged for 3 h. CeO_2_ NC was collected through acetone precipitation and dispersed in chloroform. 10 mL of citric acid and BMPA-mixed DMF solution was subsequently added into the prepared CeO_2_ NC solution and stirred vigorously overnight at room temperature to prepare carboxylated CeO_2_ NC. 5 mL of aminated MSN and 5 mL of carboxylated CeO_2_ NC were mixed and stirred overnight at room temperature to synthesize the MCe. 1 mg of Ce6 was then incubated with the obtained MCe solution in the presence of 1.5 mg of EDC and 1.6 mg of NHS. After overnight reaction at room temperature, the MCeC were prepared and finally dispersed in deionized water. J774 macrophages were cultured, and cell membranes were extracted using the method previously reported [37]. In brief, mouse J774 monocyte macrophages were cultured in DMEM high-sugar medium with/without LPS (50 ng/mL) for 48 h. Then, the cells were digested with 2 mM EDTA solution followed by centrifugation, and cell precipitate was dispersed in membrane protein buffer solution. After 15 min ice bath, the cell suspension was frozen in a liquid nitrogen tank for 5 min and then thawed at room temperature. Freeze-thawed cells were then centrifuged at 3200 g for 15 min to remove large cell debris, and the collected supernatant was subsequently centrifuged at 20000 g for 15 min to obtain the M*Φ* (n-M*Φ* and s-M*Φ*) and finally disperse in PBS. The obtained M*Φ* was extruded into vesicles through 200 nm polycarbonate film and mixed with the MCeC and ultrasonic for 5 min to obtain decoy nanozymes (MCeC@n-M*Φ* and MCeC@s-M*Φ*).

### 4.3. In Vitro LPS Neutralization and Proinflammatory Cytokine Sequestration

To evaluate the ability of MCeC@M*Φ* for LPS neutralization and proinflammatory cytokine sequestration in vitro, a certain concentration of LPS (25 ng), TNF-*α* (25 pg), IL-1*β* (50 pg), and IL-6 (15 pg) solutions was incubated with the two decoy nanozymes (MCeC@n-M*Φ* and MCeC@s-M*Φ*) with different concentrations (0, 25, 50, 100, and 200 *μ*g/mL of Ce element) for 30 min, respectively. Then, decoy nanozymes were removed by centrifugation, and the content of residual LPS and proinflammatory cytokines (TNF-*α*, IL-1*β*, and IL-6) in solution was measured using the corresponding ELISA detection kit of LPS and proinflammatory cytokines. The rate (*R*) of LPS neutralization or proinflammatory cytokine sequestration of MCeC@M*Φ* was calculated based on the following equation: *R* = (1 − (*C*_*r*_/*C*_*o*_))%. In this equation, *C*_*r*_ represents the concentration of residual LPS or proinflammatory cytokines after neutralization/sequestration, and *C*_*o*_ represents the original concentration of LPS or proinflammatory cytokines before neutralization/sequestration.

### 4.4. Analysis of the Secretion of Proinflammatory Factors by Macrophage Cells

To assess the performance of decoy nanozymes to inhibit the secretion of proinflammatory cytokines by macrophage cells upon LPS stimulation, two different experimental paths were carried out. In the experimental path I, J774 macrophages were treated with LPS (50 ng/mL) and decoy nanozymes (MCeC@n-M*Φ* and MCeC@s-M*Φ*, 200 *μ*g/mL of Ce element) at the same time, and the concentrations of proinflammatory cytokines (TNF-*α*, IL-1*β*, and IL-6) secreted by J774 macrophages after 12 h treatment were measured using the corresponding ELISA detection kit of proinflammatory cytokines. In the experimental path II, J774 cells were first stimulated by LPS (50 ng/mL) for 12 h and then treated by decoy nanozymes (MCeC@n-M*Φ* and MCeC@s-M*Φ*, 200 *μ*g/mL of Ce element). The concentration of proinflammatory cytokines (TNF-*α*, IL-1*β*, and IL-6) secreted by J774 macrophages after 1 h treatment was measured using the corresponding ELISA detection kit of proinflammatory cytokines.

### 4.5. Polarization of Macrophage M1 Phenotype

To investigate the effect of decoy nanozymes on the polarization of M1 macrophage, J774 macrophages were first induced into M1 phenotype by LPS (50 ng/mL) and then were treated by decoy nanozymes (MCeC@n-M*Φ* and MCeC@s-M*Φ*, 200 *μ*g/mL of Ce element) for 12 h. The polarization of J774 M1 phenotype to M2 phenotype upon decoy nanozymes treatment was evaluated by analyzing the secretion of anti-inflammatory cytokine of IL-10 using the ELISA detection kit.

### 4.6. SOD-Like Activity of Decoy Nanozymes

The SOD-like activity of MCeC@M*Φ* to scavenge ∙O_2_^–^ was evaluated by a SOD test kit containing xanthine, xanthine oxidase (XO), and WST-1 tetrazolium dye. In principle, ∙O_2_^–^ generated by XO-catalyzed oxidation of xanthine will react with WST-1 tetrazolium dye to produce formazan (yellow color with an absorbance around 450 nm), and this reaction will be inhibited in the presence of MCeC@M*Φ* due to the scavenging of ∙O_2_^–^. In our experiments, the mixed solution of xanthine, XO, and WST-1 tetrazolium dye was first incubated with MCeC@M*Φ* with different concentrations (0, 25, 50, 100, and 200 *μ*g/mL of Ce element) for 20 min, and the SOD-like activity (ASOD) of MCeC@M*Φ* was calculated based on the following equation: *A*_SOD_ = (1 − (*A*_test_/*A*_control_))%. In this equation, *A*_test_ represents the absorbance of reaction solution of xanthine, XO, and WST-1 tetrazolium dye and MCeC@M*Φ* at the wavelength of 450 nm, and *A*_control_ represents the absorbance of mixed solution of xanthine, XO, and WST-1 tetrazolium dye in the absence of MCeC@M*Φ* at the wavelength of 450 nm.

### 4.7. CAT-Like Activity of Decoy Nanozymes

The CAT-like activity of MCeC@M*Φ* to scavenge H_2_O_2_ was evaluated by a CAT test kit containing H_2_O_2_ and ammonium molybdate. In principle, H_2_O_2_ will react with ammonium molybdate to form a complex with an absorbance around 405 nm, and this reaction will be inhibited in the presence of MCeC@M*Φ* due to the scavenging of H_2_O_2_. In our experiments, H_2_O_2_ was first incubated with MCeC@M*Φ* with different concentrations (0, 25, 50, 100, and 200 *μ*g/mL of Ce element) for 30 min, and ammonium molybdate was then added and reacted for 5 min. The CAT-like activity (ACAT) of MCeC@M*Φ* was calculated based on the following equation: *A*_CAT_ = (1 − (*A*_test_/*A*_control_))%*A*_*SOD*_ = (1 − (*A*_test_/*A*_control_))%. In this equation, *A*_test_ represents the absorbance of reaction solution of H_2_O_2_, ammonium molybdate, and MCeC@M*Φ* at the wavelength of 405 nm, and *A*_control_ represents the absorbance of mixed solution of H_2_O_2_ and ammonium molybdate in the absence of MCeC@M*Φ* at the wavelength of 405 nm. In addition, the generation of O_2_ during the scavenging of H_2_O_2_ was analyzed to further prove the CAT-like activity of MCeC@M*Φ*. In brief, 10 mM of H_2_O_2_ was incubated with MCeC@M*Φ* (200 *μ*g/mL of Ce element), and the generation of O_2_ in the mixed solution was detected by a portable oxygen meter within 180 s. The materials of MSN, CeO_2_ NC, Ce6, and M*Φ* were used as the control.

### 4.8. HORAC Activity of Decoy Nanozymes

The HORAC activity of MCeC@M*Φ* to scavenge ∙OH was evaluated by a HORAC test kit containing H_2_O_2_, ferrous ion, and Griess reagent. In principle, ∙OH generated by ferrous ion-catalyzed Fenton reaction of H_2_O_2_ will react with Griess reagent to produce purple-red oxide (with an absorbance around 560 nm), and this reaction will be inhibited in the presence of MCeC@M*Φ* due to the scavenging of ∙OH. In our experiments, H_2_O_2_ was first incubated with the mixed solution of ferrous ion and MCeC@M*Φ* with different concentrations (0, 25, 50, 100, and 200 *μ*g/mL of Ce element) for 2 min, and then, Griess reagent was added and reacted at room temperature for 20 min. The HORAC activity of MCeC@M*Φ* was calculated based on the following equation: *A*_HORAC_ = (1 − (*A*_test_/*A*_control_))%. In this equation, *A*_test_ represents the absorbance of reaction solution of H_2_O_2_, ferrous ion, Griess reagent, and MCeC@M*Φ* at the wavelength of 560 nm, and *A*_control_ represents the absorbance of mixed solution of H_2_O_2_, ferrous ion, and Griess reagent in the absence of MCeC@M*Φ* at the wavelength of 560 nm.

### 4.9. The Mitigation of ROS in Hyperactive Macrophages

The intracellular ROS mitigation performance of MCeC@M*Φ* was evaluated in J774 macrophage cells with ROS hyperactivity state. Briefly, J774 macrophage cells were first stimulated by Rosup agent to produce high level of intracellular ROS and then treated by MCeC@M*Φ* for 12 h. Dichlorofluorescein (DCF, a fluorescent marker generated from dichlorodihydrofluorescein dye by ROS) staining was subsequently carried out to quantitatively assess the ROS level in J774 macrophages with the help of laser confocal fluorescence microscopy and fluorescence spectrometer.

### 4.10. Bacteria Culture and Antimicrobial Experiments

Multi-drug-resistant bacteria of *Escherichia coli* (*E. coli*) (ATCC BAA-3049) were used in our experiments. MDR *E. coli* were cultured in lysogeny broth (LB) medium and harvested at the exponential growth phase before use. For antimicrobial experiments, 10^6^ CFU of MDR bacteria was incubated with MCeC@M*Φ* (200 *μ*g/mL of Ce element) under the condition of laser irradiation (660 nm, 0.8 W/cm^2^, 5 min), and the antimicrobial performance was evaluated by the bacterial growth curve analysis, bacterial ROS determination, live/dead bacterial staining assay, and SEM-based bacterial morphology investigation (see the experimental details in Supporting Information).

### 4.11. MDR Biofilm Eradication

The capability of MCeC@M*Φ* for MDR biofilm formation inhibition and destruction was investigated by crystal violet staining. For MDR biofilm formation inhibition, 10^6^ CFU of MDR *E. coli* suspensions were mixed with MCeC@M*Φ* (200 *μ*g/mL of Ce element) in 96-well plates and then irradiated by a 660 nm laser (0.8 W/cm^2^) for 5 min. After 2-day incubation, the plates were gently washed by PBS, and crystal violet ethanol solution was added and reacted for 15 min. The plates after crystal violet staining were then imaged by camera, and the corresponding absorbance of staining solution at 590 nm was measured to indicate the extent of biofilm formation. For mature MDR biofilm destruction, 10^6^ CFU of MDR *E. coli* suspensions were added into 96-well plates and grown 2 days to form integrated biofilm. Then, MCeC@M*Φ* (200 *μ*g/mL of Ce element) was added onto the surface of mature MDR *E. coli* biofilm followed by laser irradiation (660 nm, 0.8 W/cm^2^, 5 min), and then, crystal violet ethanol solution was added and reacted for 15 min. Finally, the plates after crystal violet staining were imaged by camera, and the corresponding absorbance of staining solution at 590 nm was measured to indicate the extent of biofilm destruction.

### 4.12. ROS Decay after PDT

The decay of ROS in MCeC@M*Φ* after irradiation was investigated using SOSG. In brief, MCeC@M*Φ* (200 *μ*g/mL of Ce element) was mixed with SOSG (50 *μ*M) and irradiated (660 nm laser, 0.8 W/cm^2^) for 5 min. The fluorescence spectra of the solution were collected at different time points after laser irradiation (0, 5, and 10 min) to indicate the level of residual ROS after PDT. The decay of ROS in Ce6 after irradiation (660 nm laser, 0.8 W/cm^2^, 5 min) was used as the control.

### 4.13. Mouse Model of MDR Bacterial Sepsis

Female mice (BALB/c, 6 weeks) were purchased from Jinan Pengyue Biotechnology Co., Ltd., and allowed to adapt in the laboratory for one week before experiment. All animal experiments were carried out in compliance with the protocols approved by the Shandong University Laboratory Animal Center. In our experiments, the mouse model of MDR bacterial sepsis was established by inoculating lethal doses of MDR *E. coli* (2 × 10^8^ CFU) through intraperitoneal (*i.p.*) injection to trigger aberrant inflammatory response.

### 4.14. In Vivo MDR Bacterial Sepsis Treatment

The mice with MDR bacterial sepsis were divided into four treatment groups including PBS (positive control), MCeC, MCeC@M*Φ*, and MCeC@M*Φ* plus irradiation (MCeC@M*Φ*/Ir), while the group of normal healthy mice was used as the negative control. Each treatment group contained 10 mice. To carry out *in vivo* bacterial sepsis treatment, septic mice were treated by intraperitoneally injecting materials (PBS, MCeC, MCeC@M*Φ*, or MCeC@M*Φ*/Ir) at two time points (0.5, and 12 h) after bacterial inoculation. At the 24^th^ h of bacterial inoculation, 4 mice in each treatment group were sacrificed, and the amount of typical proinflammatory factors (TNF-*α*, IL-6, and IL-1*β*) in blood and peritoneal exudate, protein permeability in peritoneal exudate, bacterial burden in blood, peritoneal exudate and major organs (kidney, spleen, and liver), and organ histological condition (H&E staining) were measured and analyzed. In addition, the survival of septic mice in different treatment groups within 120 h after bacterial inoculation was quantitatively analyzed to evaluate the actual therapeutic ability of MCeC@M*Φ* for *in vivo* MDR bacterial sepsis treatment.

### 4.15. Statistical Analysis

Data are expressed as mean ± standard deviation. Student's two-tailed *t* tests was performed for statistical analysis, n.s. indicates *P* > 0.5, ∗/# indicates *P* < 0.05, ∗∗/## indicates *P* < 0.01, and ∗∗∗/### indicates *P* < 0.001.

## Data Availability

The data that support the findings of this study are available within the article and its supplementary materials. Raw data are available from the corresponding authors on reasonable request.

## Supplementary Materials

Supplementary Materials Experimental section. Supplementary figures. Figure S1: XRD spectra of MSN, CeO_2_ NC, and MCe. Figure S2: FTIR spectra of MSN, CeO_2_ NC, and MCe. Figure S3: EDX mapping of MCeC@M*Φ* and quantitative element analysis. Figure S4: FTIR spectrum of MC. Figure S5: release curves of Ce6 from MCeC and Ce6/MCe. Figure S6: standard absorption curve of Ce6. Figure S7: pore size distribution of MSN and MCec. Figure S8: zeta potentials of MSN, MCe, MCeC, M*Φ*, and MCeC@M*Φ*. Figure S9: hydrodynamic diameters of MSN, MCe, MCeC, M*Φ*, and MCeC@M*Φ*. Figure S10: cell antiproliferation assay of MCeC@M*Φ*. Figure S11: schematic illustration of decoy nanozyme-based LPS/proinflammatory cytokine neutralization/sequestration. Figure S12: LPS binding amounts of decoy nanozymes. Figure S13: the binding amounts of TNF-*α*, IL-1*β*, and IL-6 of decoy nanozymes. Figure S14: LPS neutralization and TNF-*α*/IL-1*β*/IL-6 sequestration rates of decoy nanozymes. Figure S15: the secretion of anti-inflammatory cytokine of IL-10 by J774 M1 phenotype upon the treatment of decoy nanozymes. Figure S16: the expression of M2 macrophage marker of Arg-1 by J774 M1 phenotype upon the treatment of decoy nanozymes. Figure S17: XPS spectrum of MCeC@M*Φ*. Figure S18: O_2_ generation in the solution of H_2_O_2_ after incubation with MCeC@M*Φ*. Figure S19: the performance of MCeC@M*Φ* and MCeC@M*Φ*/Ir for biofilm formation. Figure S20: the performance of MCeC@M*Φ* for the elimination of PDT-aggravated inflammation. Figure S21: bacterial burden evaluation in septic mice under different treatment conditions. Figure S22: histological analysis of the liver and kidney of septic mice under different treatment conditions. Figure S23: histological analysis of spleen of septic mice under different treatment conditions. Figure S24: the distribution of Ce element in the organs of healthy mice intraperitoneally injected with MCeC@M*Φ* at different time points. Figure S25: blood biochemistry data of healthy mice on the 5^th^ day of MCeC@M*Φ* postinjection. Figure S26: blood routine data of healthy mice on the 5^th^ day of MCeC@M*Φ* postinjection. Figure S27: H&E staining images of major organs of healthy mice on the 5^th^ day of MCeC@M*Φ* postinjection.
