# Supplementary material for: Decoy Nanozymes Enable Multitarget Blockade of Proinflammatory Cascades for the Treatment of Multi-Drug-Resistant Bacterial Sepsis
Source: Research (Wash D C). 2022 Sep 26;2022:9767643. doi: 10.34133/2022/9767643 (PMC9534579; doi:10.34133/2022/9767643)
Supplement: Supplementary Materials — Experimental section. Supplementary figures. Figure S1: XRD spectra of MSN, CeO2 NC, and MCe. Figure S2: FTIR spectra of MSN, CeO2 NC, and MCe. Figure S3: EDX mapping of MCeC@MΦ and quantitative element analysis. Figure S4: FTIR spectrum of MC. Figure S5: release curves of Ce6 from MCeC and Ce6/MCe. Figure S6: standard absorption curve of Ce6. Figure S7: pore size distribution of MSN and MCec. Figure S8: zeta potentials of MSN, MCe, MCeC, MΦ, and MCeC@MΦ. Figure S9: hydrodynamic diameters of MSN, MCe, MCeC, MΦ, and MCeC@MΦ. Figure S10: cell antiproliferation assay of MCeC@MΦ. Figure S11: schematic illustration of decoy nanozyme-based LPS/proinflammatory cytokine neutralization/sequestration. Figure S12: LPS binding amounts of decoy nanozymes. Figure S13: the binding amounts of TNF-α, IL-1β, and IL-6 of decoy nanozymes. Figure S14: LPS neutralization and TNF-α/IL-1β/IL-6 sequestration rates of decoy nanozymes. Figure S15: the secretion of anti-inflammatory cytokine of IL-10 by J774 M1 phenotype upon the treatment of decoy nanozymes. Figure S16: the expression of M2 macrophage marker of Arg-1 by J774 M1 phenotype upon the treatment of decoy nanozymes. Figure S17: XPS spectrum of MCeC@MΦ. Figure S18: O2 generation in the solution of H2O2 after incubation with MCeC@MΦ. Figure S19: the performance of MCeC@MΦ and MCeC@MΦ/Ir for biofilm formation. Figure S20: the performance of MCeC@MΦ for the elimination of PDT-aggravated inflammation. Figure S21: bacterial burden evaluation in septic mice under different treatment conditions. Figure S22: histological analysis of the liver and kidney of septic mice under different treatment conditions. Figure S23: histological analysis of spleen of septic mice under different treatment conditions. Figure S24: the distribution of Ce element in the organs of healthy mice intraperitoneally injected with MCeC@MΦ at different time points. Figure S25: blood biochemistry data of healthy mice on the 5th day of MCeC@MΦ postinjection. Figure S26: b [file 9767643.f1.docx]

**Supporting Information**

**Decoy Nanozymes Enable Multi-Target Blockade of Pro-Inflammatory Cascades for the Treatment of Multidrug-Resistant Bacterial Sepsis**

Xuancheng Du^1,§^, Mingzhen Zhang^2,§^, Huiting Zhou^3,§,^, Weijie Wang^1^, Chengmei Zhang^4^, Lei Zhang^5^, Yuanyuan Qu^1^, Weifeng Li^1^*, Xiangdong Liu^1^, Mingwen Zhao^1^, Kangsheng Tu^6^*, and Yong-Qiang Li^1,7,8^*

^1^ Institute of Advanced Interdisciplinary Science, School of Physics, Shandong University, Jinan 250100, China.

^2^ School of Basic Medical Sciences, Xi’an Key Laboratory of Immune Related Diseases, Xi’an Jiaotong University, Xi’an 710061, China.

^3^ Institute of Pediatric Research, Children’s Hospital of Soochow University, Suzhou 215025, China.

^4^ Laboratory Animal Center of Shandong University, Jinan 250012, China.

^5^ Department of Critical Care Medicine, the First Affiliated Hospital of Xi’an Jiaotong University, Xi’an 710061, China.

^6^ Department of Hepatobiliary Surgery, the First Affiliated Hospital of Xi’an Jiaotong University, Xi’an 710061, China.

^7^ Suzhou Research Institute, Shandong University, Suzhou 215123, China.

^8^ College of Chemistry, Chemical Engineering and Materials Science, Shandong Provincial Key Laboratory of Clean Production of Fine Chemicals, Shandong Normal University, Jinan 250014, China.

^§^ These authors contributed equally to this work.

1. Experimental

*Characterization of Decoy Nanozymes*

The morphology and size of MCeC@MΦ were observed by transmission electron microscope, while the hydrodynamic size and zeta potential were monitored by Nanosizer. The UV-vis absorption spectrum, infrared spectrum and fluorescence spectrum of MCeC@MΦ were characterized by UV-vis spectrophotometer, Fourier transform infrared spectrometer, and transient steady-state fluorescence spectrometer, respectively. The X-ray diffraction pattern and photoelectron spectrum of MCeC@MΦ were recorded by in-situ powder X-ray diffractometer and photoelectron spectroscopy, respectively. The N_2_ adsorption-desorption isotherm and pore size distribution of MCeC@MΦ were measured by multi-station extended specific surface area and pore size analyzer. The concentration of cerium element in MCeC@MΦ was quantified by inductively coupled plasma mass spectrometry. The expression of target proteins (TNF-R1, TLR-4, IL-1R1, IL-6Rα) in MCeC@MΦ were analyzed by Western blot.

*In Vitro Biocompatibility Assay*

The biocompatibility of MCeC@MΦ was determined by MTT assay using human umbilical vein endothelial cells (HUVEC). In brief, HUVEC cells were seeded into a 96-well plate (8000-10000 cells/well) and cultured overnight. Then the cells were treated with MCeC@MΦ with different concentrations (0, 25, 50, 100, and 200 µg/mL of Ce element). After 24 h of culture, MTT reagent was added and the cell viability was evaluated with a microplate reader.

*Cellular Uptake of Decoy Nanozymes in Macrophage*

To investigate the cellular uptake of MCeC@MΦ in macrophage, J774 macrophage cells were incubated with MCeC@MΦ (200 µg/mL of Ce element), and imaged using laser confocal fluorescence microscopy at different incubation time points (0, 4, and 8 h) by exploiting the fluorescence of Ce6 in MCeC@MΦ.

*Bacterial Growth Curve Analysis*

10^6^ CFU of MDR *E. coli* was first mixed with MCeC@MΦ (200 µg/mL of Ce element), and then irradiated by a 660 nm laser with a power of 0.8 W/cm^2^ for 5 min, while the mixed solution of MDR *E. coli* and MCeC@MΦ without irradiation was used as the control. The OD_600_ value of the mixed solutions was measured per 1 h to plot the bacterial growth curve. At the time point of 12 h, bacteria liquid was taken for bacterial plating test.

*Determination of ROS in MDR Bacteria*

2ʹ,7ʹ-Dichlorofluorescin Diacetate (DCFH-DA) was used to determine the level of ROS in MDR bacteria. In brief, MDR *E. coli* before and after MCeC@MΦ treatment were first incubated with DCFH-DA (10 μM) for 30 min in dark, respectively, and washed three times with PBS after centrifugation. Then bacteria were imaged by a confocal fluorescence microscope, and their fluorescence spectrums were recorded using a microplate reader.

*Live/Dead Bacterial Staining Assay*

Live/dead staining assay kit was used to evaluate the viability of bacteria. In brief, MDR *E. coli* before and after MCeC@MΦ treatment were mixed with the dye solution containing SYTO 9 and propidium iodide for 30 min in the dark, and then imaged with a confocal fluorescence microscope. Live bacteria were stained by SYTO 9 with green color, while dead bacteria were stained by propidium iodide with red color due to the damage of cell membrane and wall.

*SEM-based Morphological Study of Bacteria*

The morphology of bacteria was characterized by field-emission scanning electron microscopy (FESEM). In brief, MDR *E. coli* before and after MCeC@MΦ treatment were fixed with glutaraldehyde (2.5%) in the dark for 2 h, and dehydrated by ethanol solution with different concentrations (50%, 70%, 90% and 100%) for 10 min. The dehydrated bacterial samples were dropped on silicon wafer, and imaged with FESEM after nitrogen drying and platinum coating.

*In Vivo Biocompatibility Investigation of Decoy Nanozymes*

Pharmacokinetic distribution, organ pathological examination as well as blood biochemistry assay were conducted to evaluate the *in vivo* biosafety of MCeC@MΦ. In brief, MCeC@MΦ (200 μg/mL of Ce element) was intraperitoneally injected into healthy mice, and the amount of Ce element in organs at different time points (24, 48, and 72 h) after MCeC@MΦ injection was determined by ICP-MS to investigate the pharmacokinetic distribution of MCeC@MΦ *in vivo*. In addition, major organs and blood samples were collected on the 5^th^ day of MCeC@MΦ post-injection. HE staining of organs and blood biochemistry assay were performed to demonstrate the *in vivo* biocompatibility of MCeC@MΦ. Organ pathological examination and blood biochemistry assay of healthy mice injected with PBS were used as the control.

2. Supporting figures


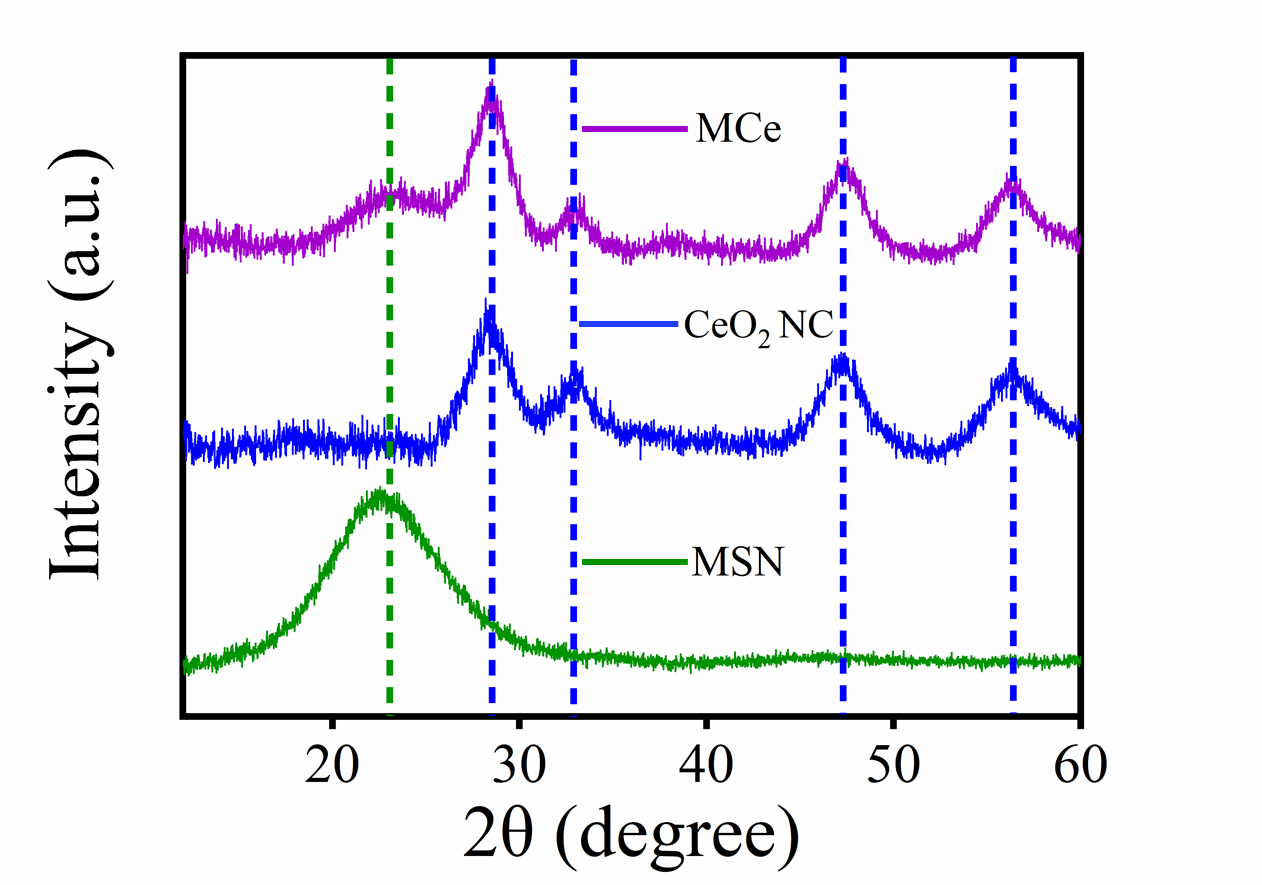


**Figure S1.** XRD spectra of MSN, CeO_2_ NC, and MCe.


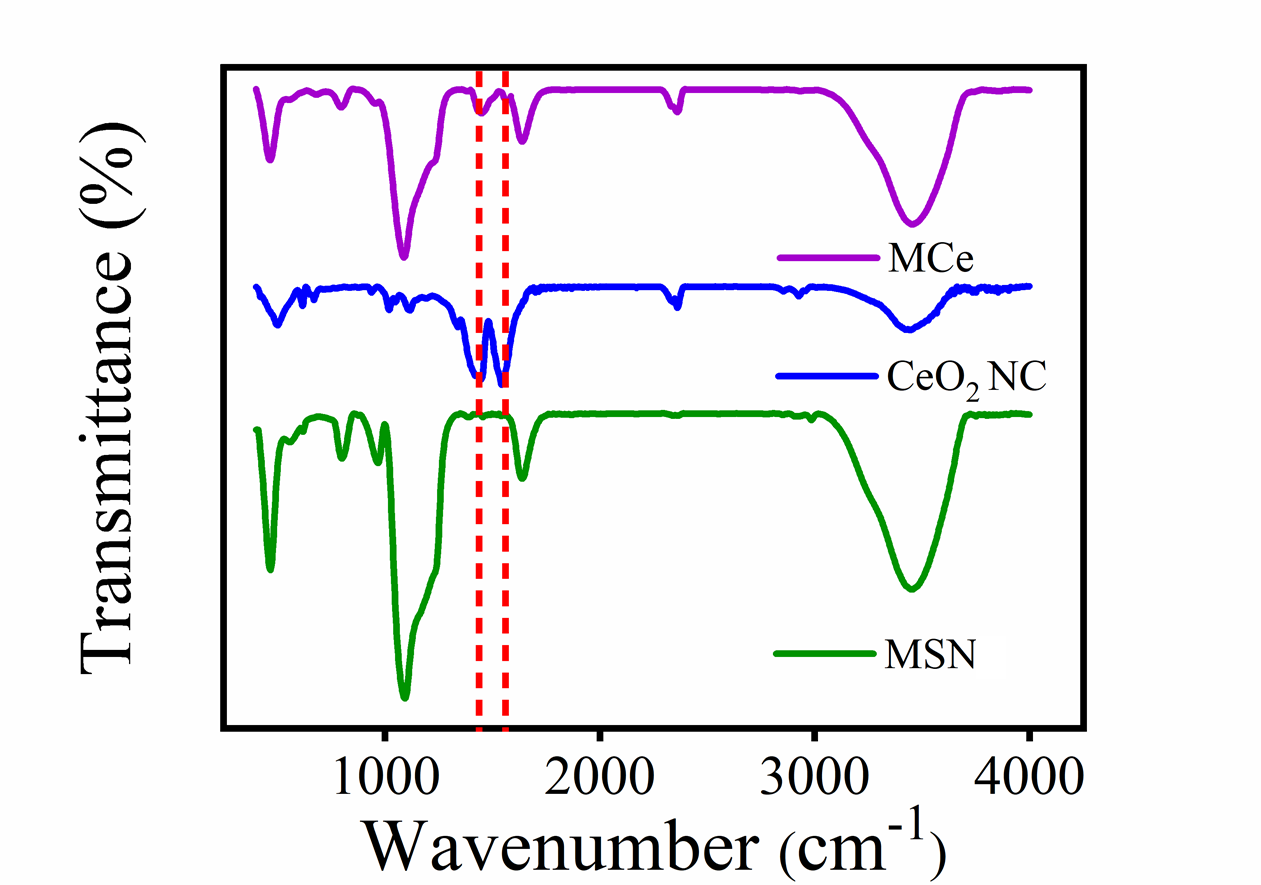


**Figure S2.** FTIR spectra of MSN, CeO_2_ NC, and MCe.


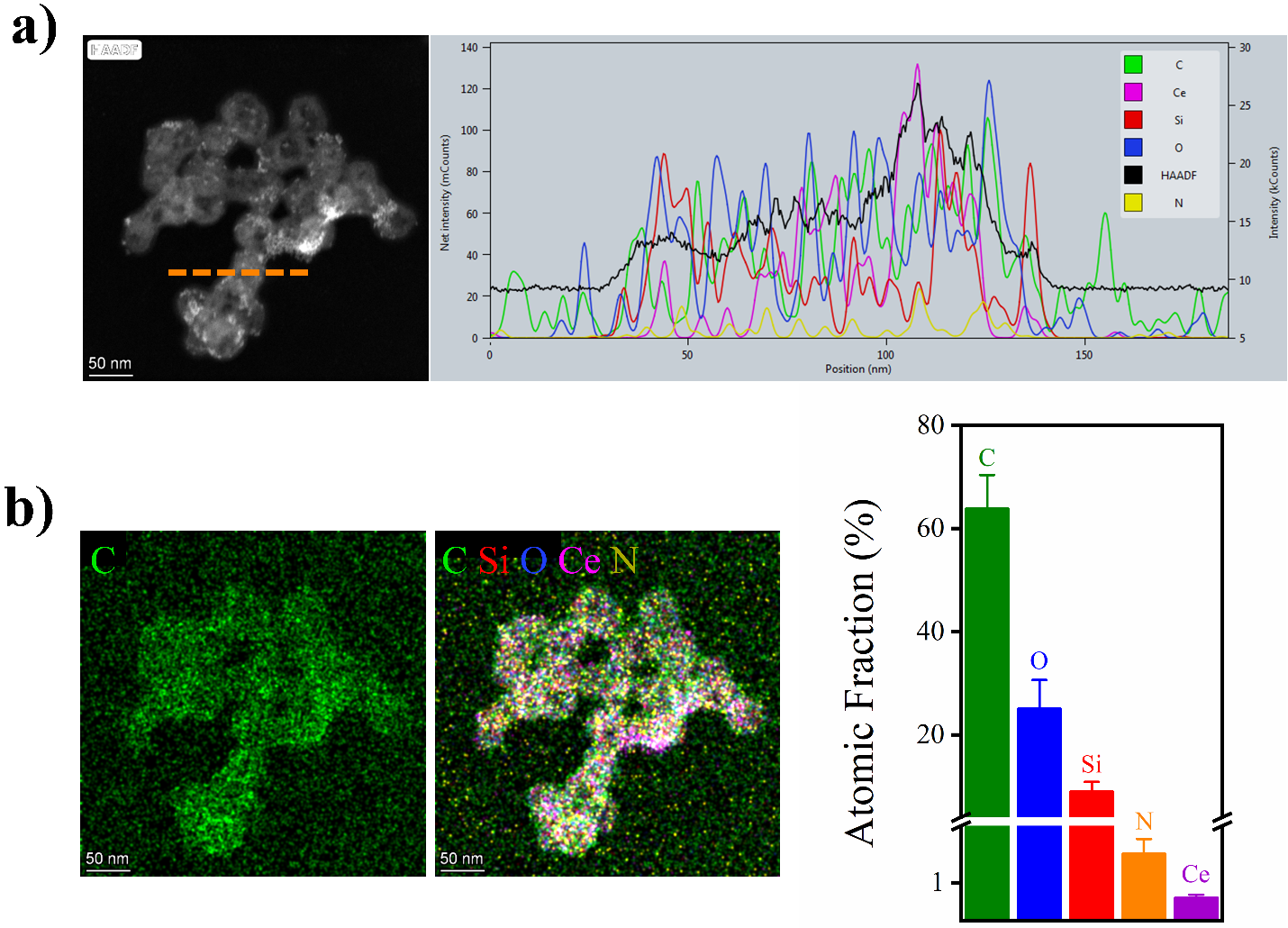


**Figure S3.** a) High-angle annular dark-field scanning TEM image and linear EDX mapping of MCeC@MΦ. b) Quantitative element analysis of MCeC@MΦ. The value of element content represents the mean of three independent experiments, and the error bars indicate the standard deviation (SD) from the mean.





**Figure S4.** FTIR spectrum of MC.


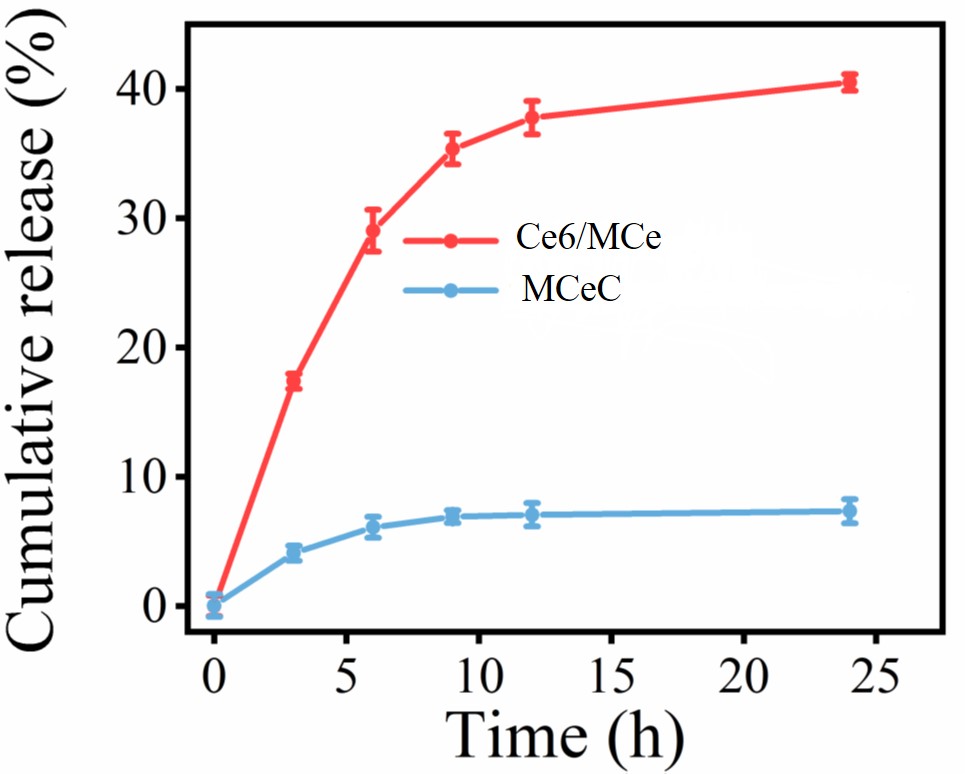


**Figure S5.** Release curves of Ce6 from MCeC and Ce6/MCe.

**

**

**Figure S6.** Standard absorption curve of Ce6.


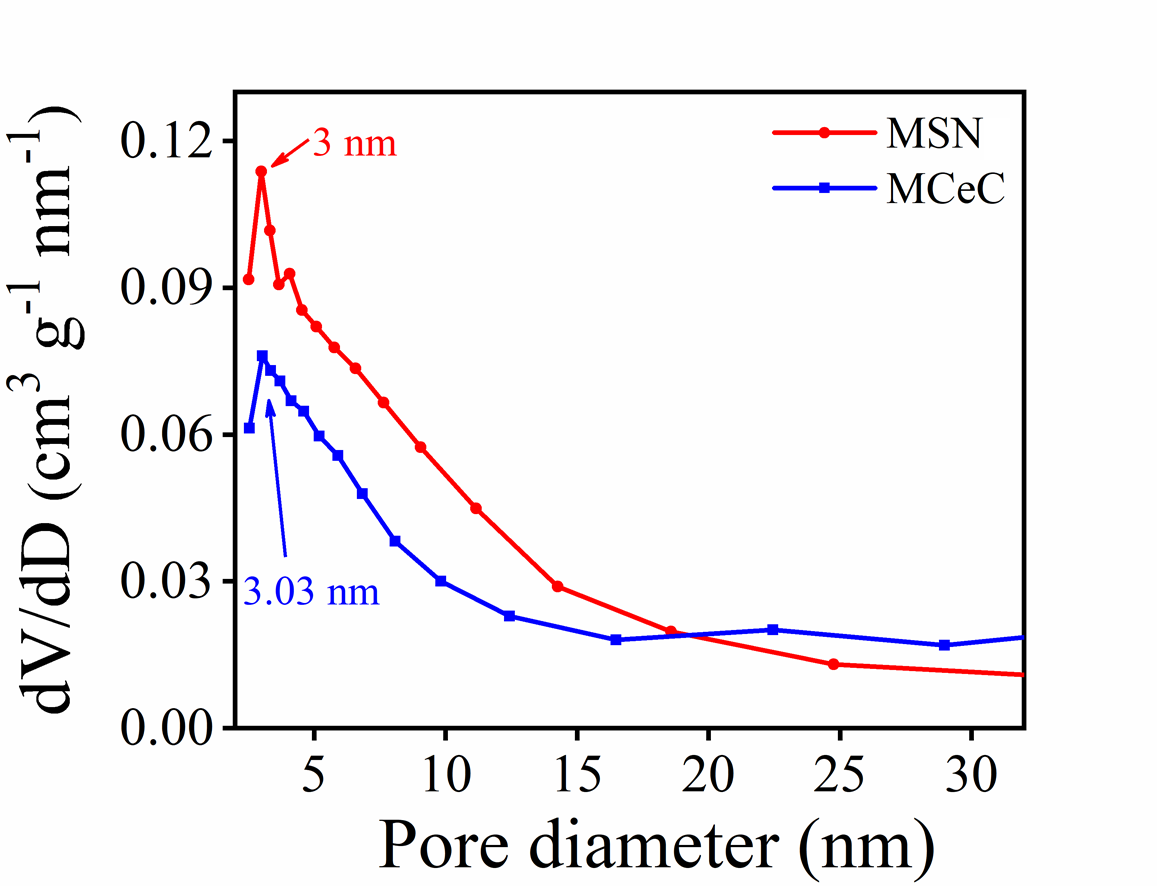


**Figure S7.** Pore size distribution of MSN and MCeC.


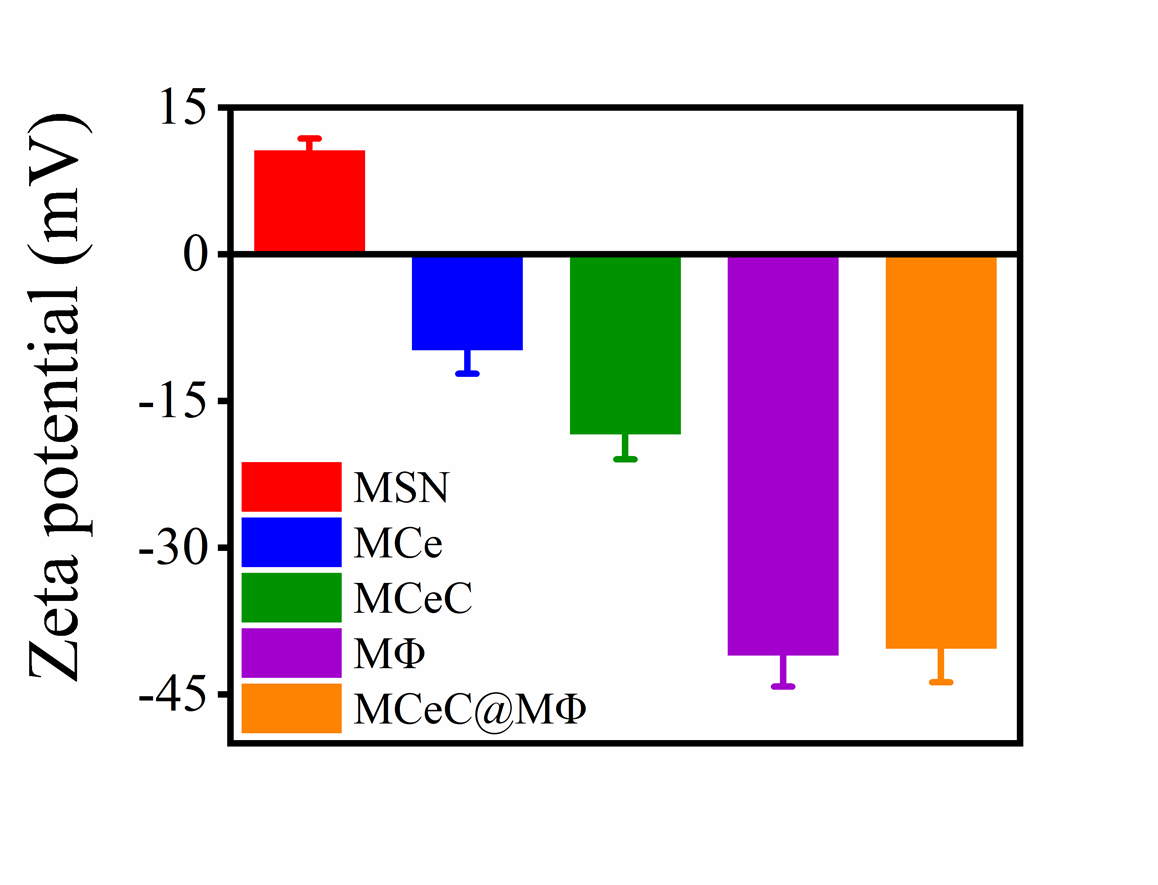


**Figure S8.** Zeta potentials of MSN, MCe, MCeC, MΦ, and MCeC@MΦ. The values of zeta potential represent the mean of three independent experiments, and the error bars indicate the SD from the mean.


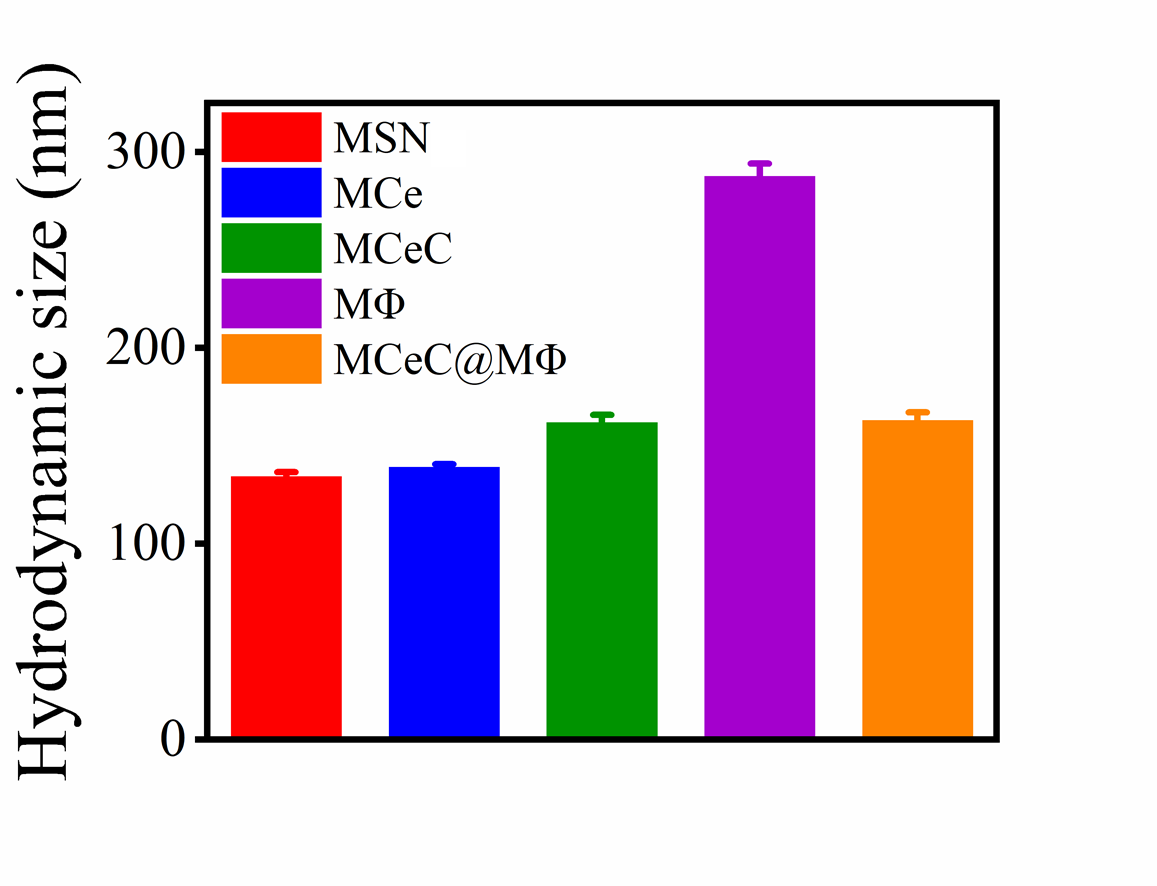


**Figure S9.** Hydrodynamic diameters of MSN, MCe, MCeC, MΦ, and MCeC@MΦ in PBS buffer. The values of hydrodynamic diameter represent the mean of three independent experiments, and the error bars indicate the SD from the mean.


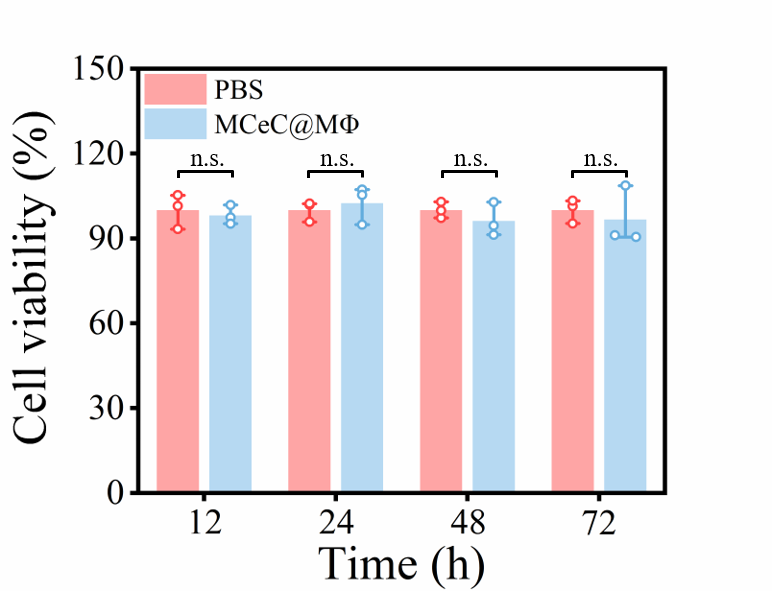


**Figure S10.** Proliferation of HUVEC cells after incubation with MCeC@MΦ for different times. The values of cell viability represent the mean of three independent experiments, and the error bars indicate the SD from the mean. ^n.s.^*P* > 0.05.


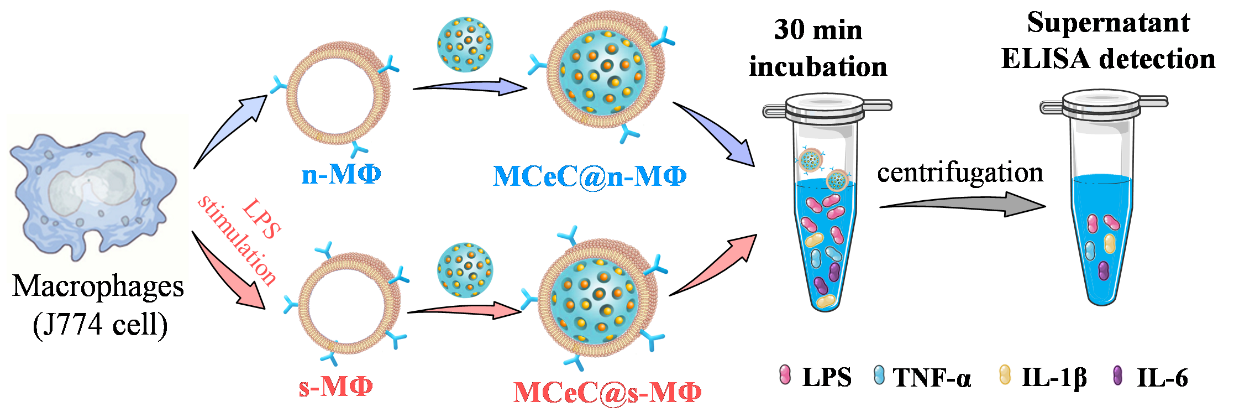


**Figure S11.** Schematic illustration of experimental procedures for endotoxin (LPS) and pro-inflammatory cytokines (TNF-α, IL-6 and IL-1β) binding and neutralization/sequestration based on decoy nanozymes (MCeC@n-MΦ and MCeC@s-MΦ).


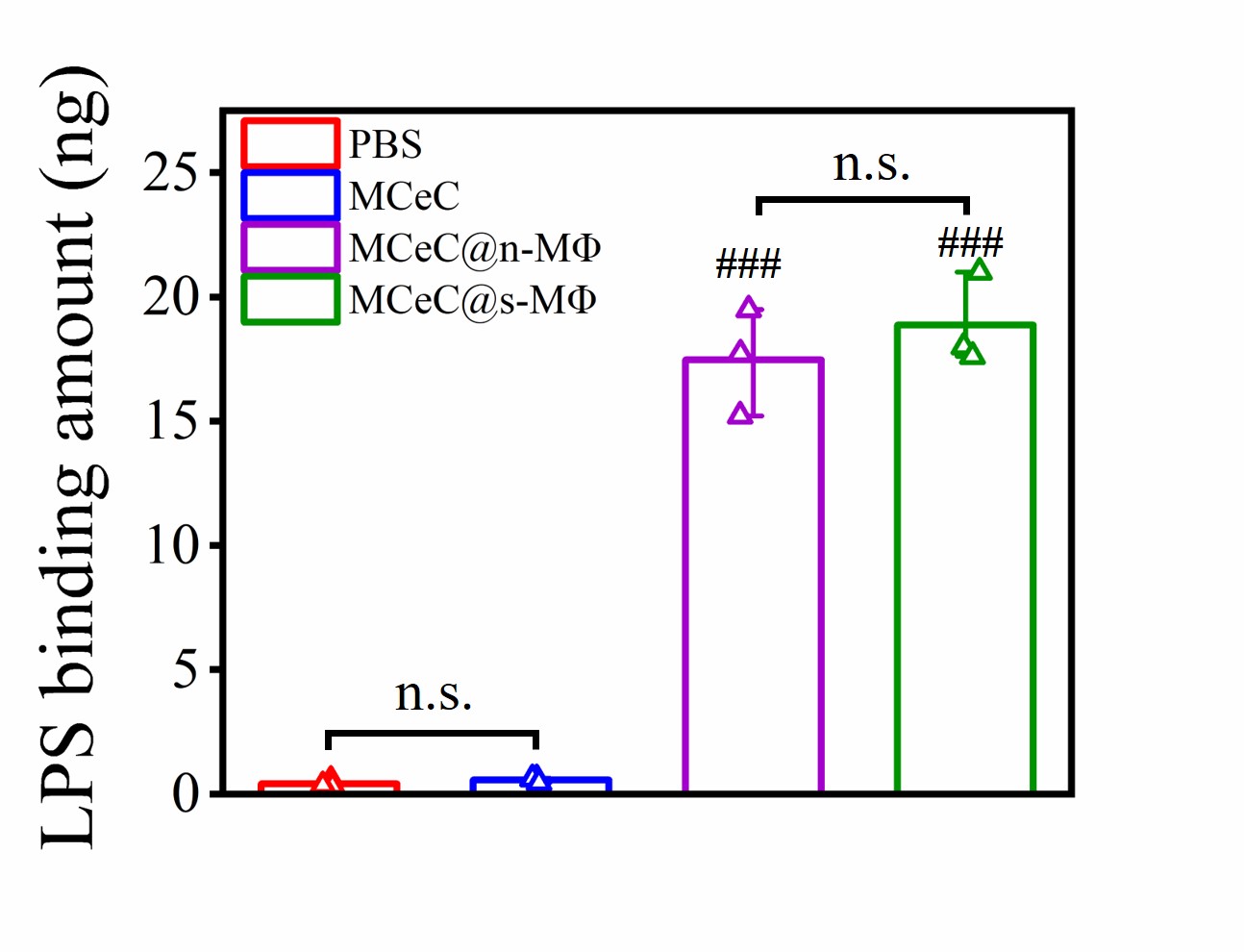


**Figure S12.** The LPS binding amounts of decoy nanozymes (MCeC@n-MΦ and MCeC@s-MΦ). The groups of PBS and MCeC were used as the control. The concentration of MCeC@n-MΦ, MCeC@s-MΦ and MCeC used was 200 µg/mL (Ce element). The values of LPS binding amount represent the mean of three independent experiments, and the error bars indicate the SD from the mean. # indicates the contrasts between experimental groups and control (PBS). ^###^*P* < 0.001, and ^n.s.^*P* > 0.05.


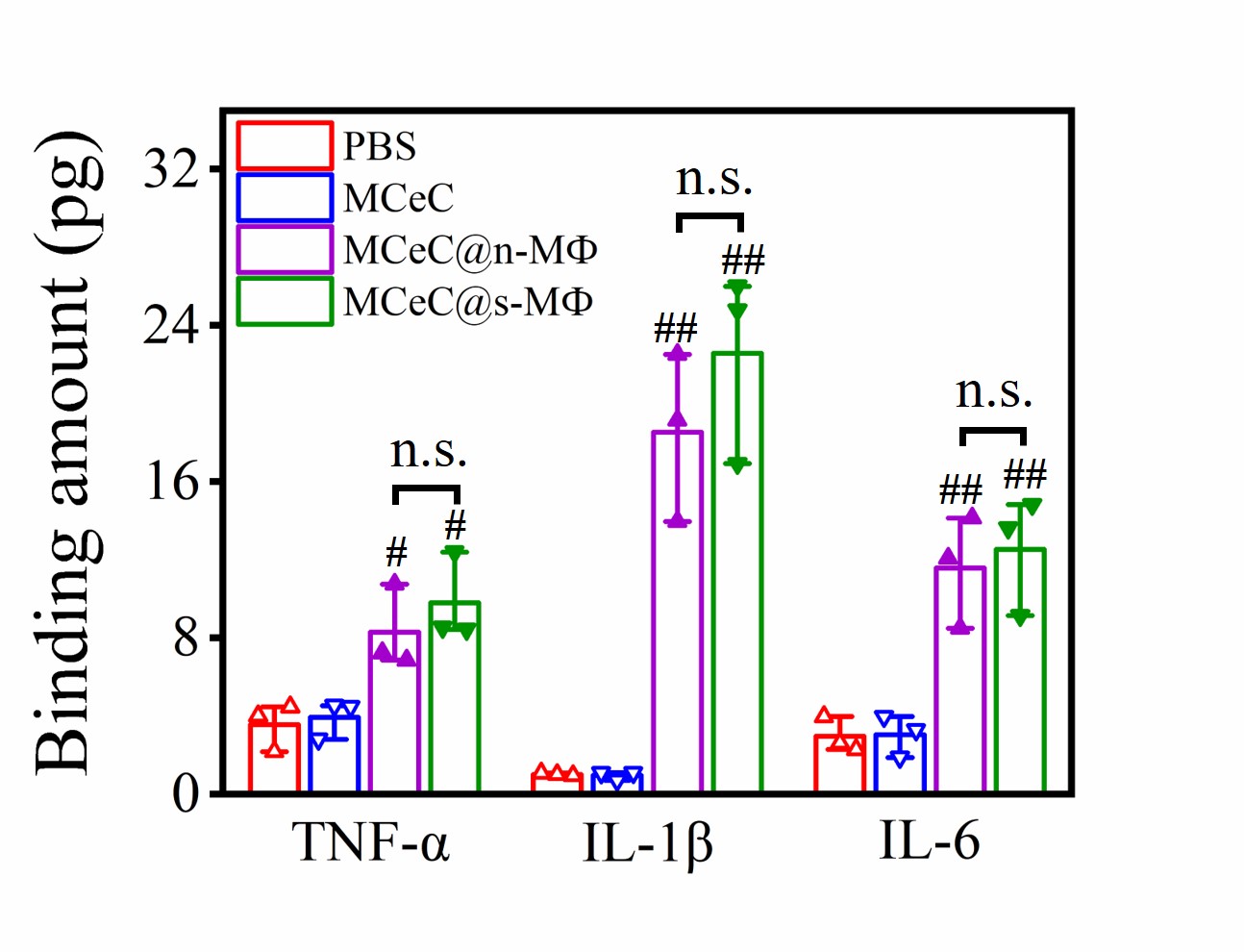


**Figure S13.** The binding amounts of TNF-α, IL-1β, and IL-6 of decoy nanozymes (MCeC@n-MΦ and MCeC@s-MΦ). The groups of PBS and MCeC were used as the control. The concentration of MCeC@n-MΦ, MCeC@s-MΦ and MCeC used was 200 µg/mL (Ce element). The values of binding amount of TNF-α, IL-1β, and IL-6 represent the mean of three independent experiments, and the error bars indicate the SD from the mean. # indicates the contrasts between experimental groups and control (PBS). ^#^*P* < 0.05, ^##^*P* < 0.01, and ^n.s.^*P* > 0.05.


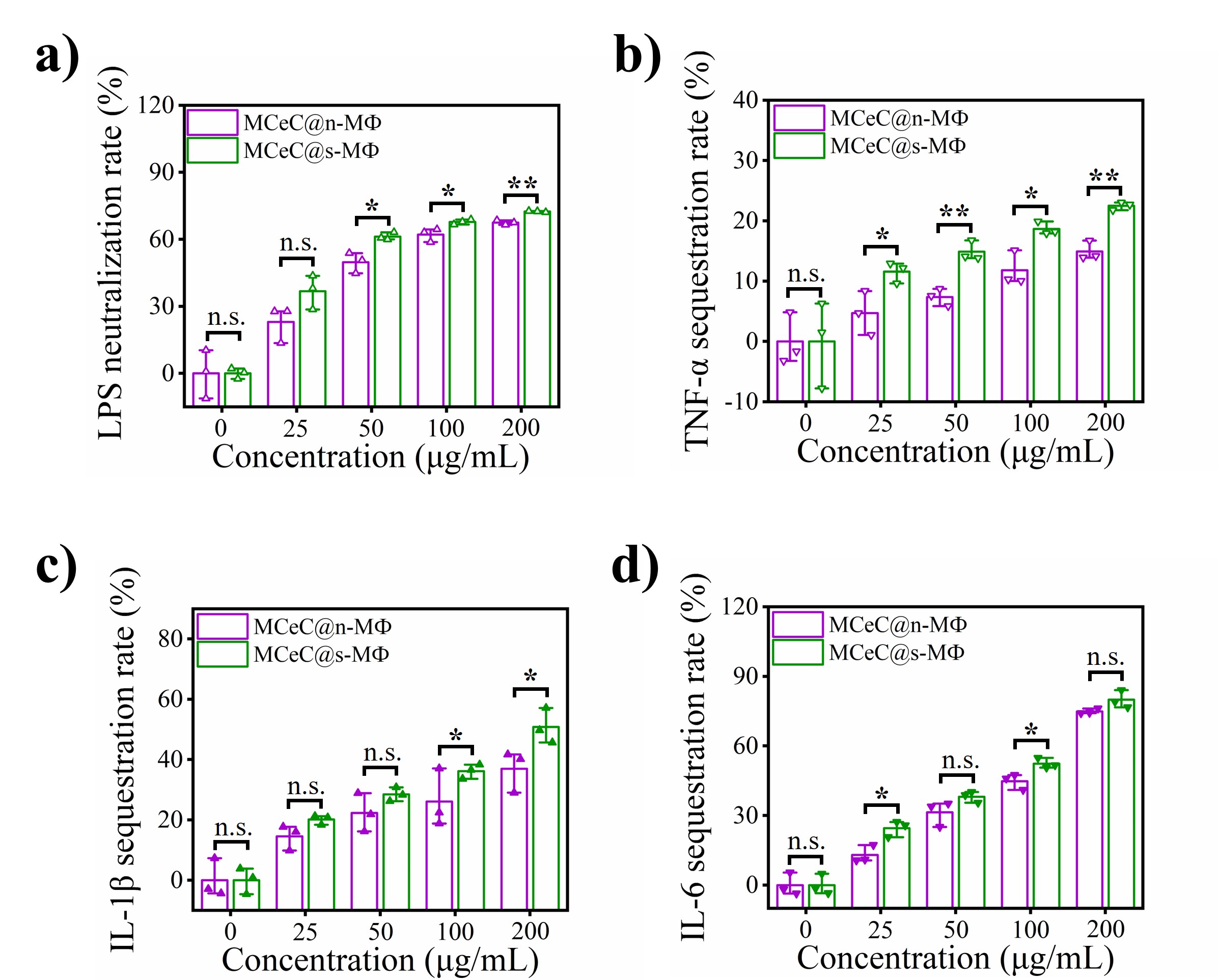


**Figure S14.** The LPS neutralization (a), TNF-α sequestration (b), IL-1β sequestration (c), and IL-6 sequestration (d) rates of decoy nanozymes (MCeC@n-MΦ and MCeC@s-MΦ) with different concentrations. The values of neutralization/sequestration rates represent the mean of three independent experiments, and the error bars indicate the SD from the mean. ^*^*P* < 0.05, ^**^*P* < 0.01, and ^n.s.^*P* > 0.05.


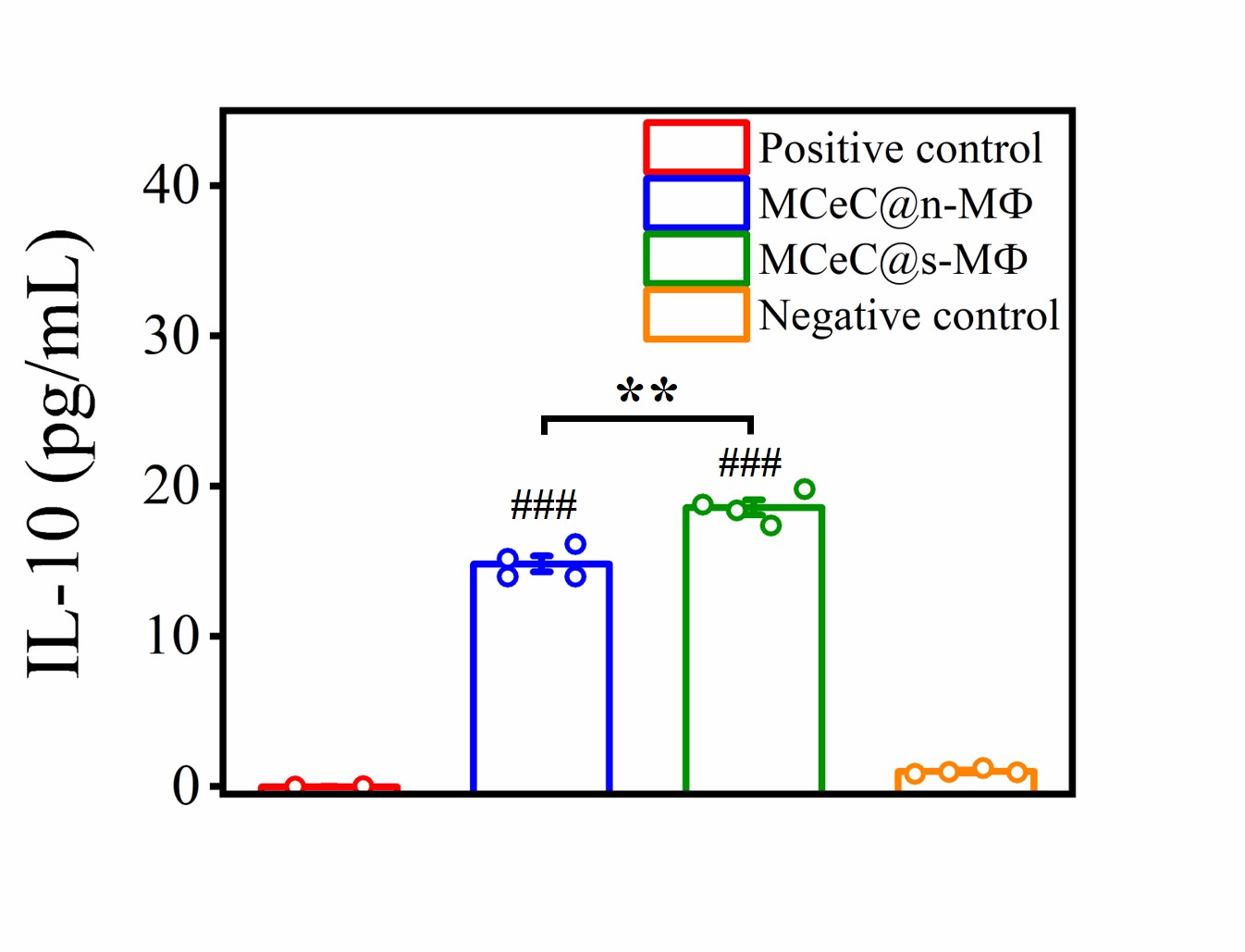


**Figure S15.** The secretion of anti-inflammatory cytokine of IL-10 by J774 M1 phenotype upon the treatment of decoy nanozymes (MCeC@n-MΦ and MCeC@s-MΦ) to indicate the degree of M1/M2 polarization. The treatment group of PBS was used as the positive control, while the normal J774 macrophage without LPS stimulation was used the negative control. The values of IL-10 amount secreted represent the mean of four independent experiments, and the error bars indicate the SD from the mean. # indicates the contrasts between experimental groups and positive control. ^**^*P* < 0.01, and ^###^*P* < 0.001.

**
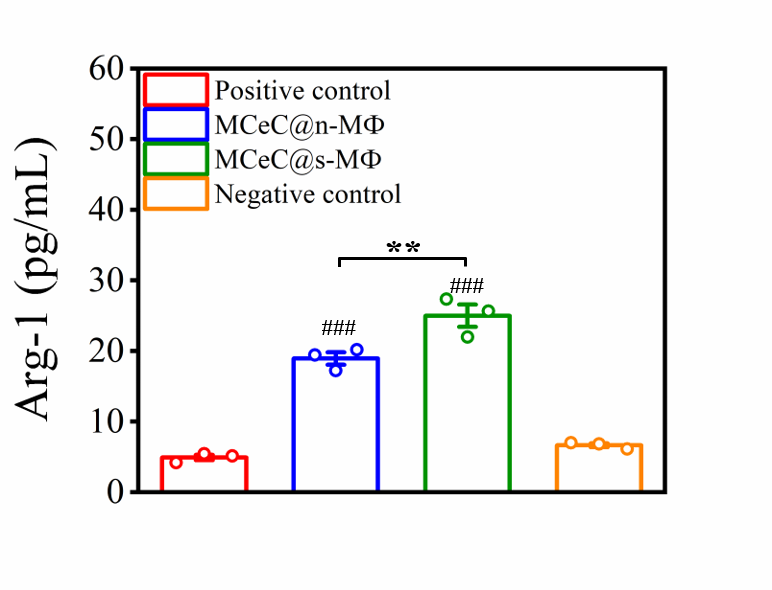
**

**Figure S16.** The expression of M2 macrophage marker of Arg-1 in J774 M1 phenotype upon the treatment of decoy nanozymes (MCeC@n-MΦ and MCeC@s-MΦ) to indicate the degree of M1/M2 polarization. The treatment group of PBS was used as the positive control, while the normal J774 macrophage without LPS stimulation was used the negative control. The values of Arg-1 amount expressed represent the mean of four independent experiments, and the error bars indicate the SD from the mean. # indicates the contrasts between experimental groups and positive control. ^**^*P* < 0.01, and ^###^*P* < 0.001.


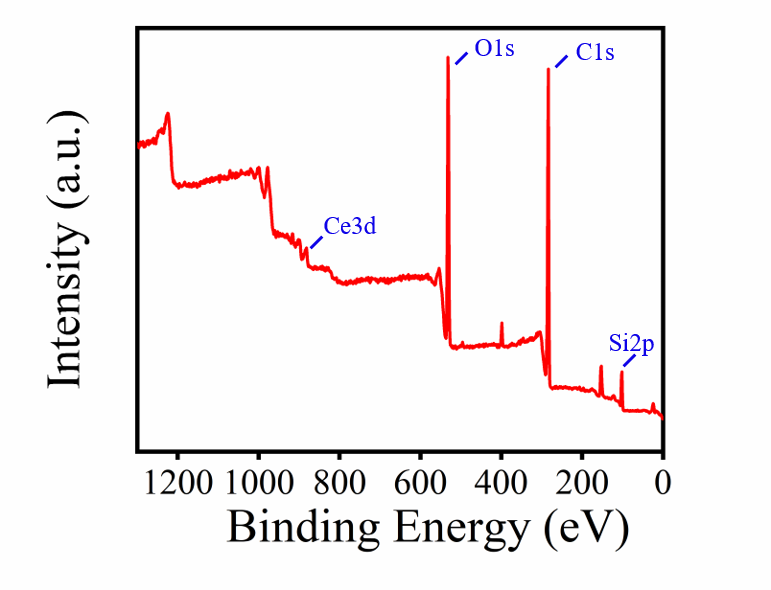


**Figure S17.** XPS spectrum of MCeC@MΦ.





**Figure S18.** The change of dissolved O_2_ concentration in the solution of H_2_O_2_ (10 mM) after incubation with MSN (1 mg/mL), CeO_2_ NC (200 µg/mL of Ce element), Ce6 (10 µg/mL), MΦ (2 mg/mL), and MCeC@MΦ (200 µg/mL of Ce element) for 180 s, respectively.


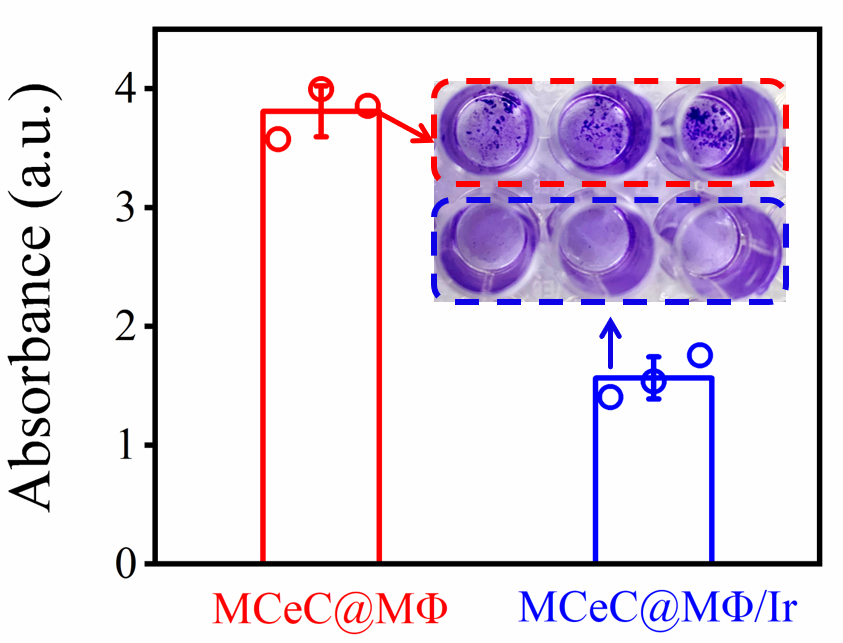


**Figure S19.** Crystal violet staining image and its corresponding absorbance for the formation of MDR *E. coli* biofilm in the presence of MCeC@MΦ and MCeC@MΦ/Ir, respectively. The values of crystal violet absorbance represent the mean of three independent experiments, and the error bars indicate the SD from the mean.


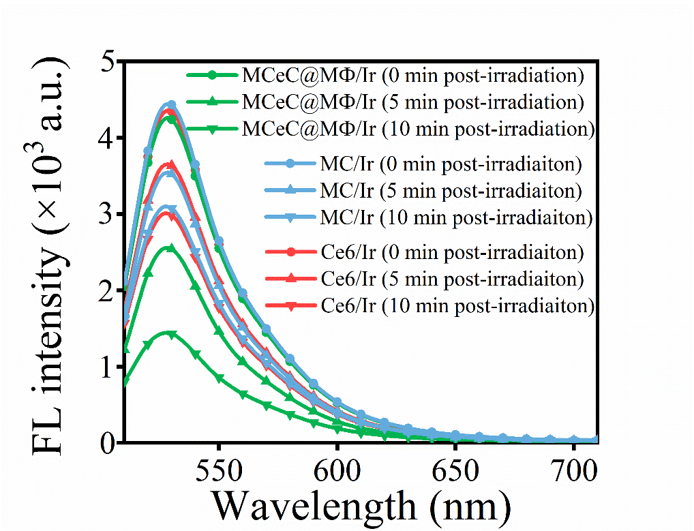


**Figure S20.** The SOSG fluorescence spectra in MCeC@MΦ, Ce6, and MC within 10 min after laser irradiation (660 nm laser, 0.8 W/cm^2^, 5 min), respectively.


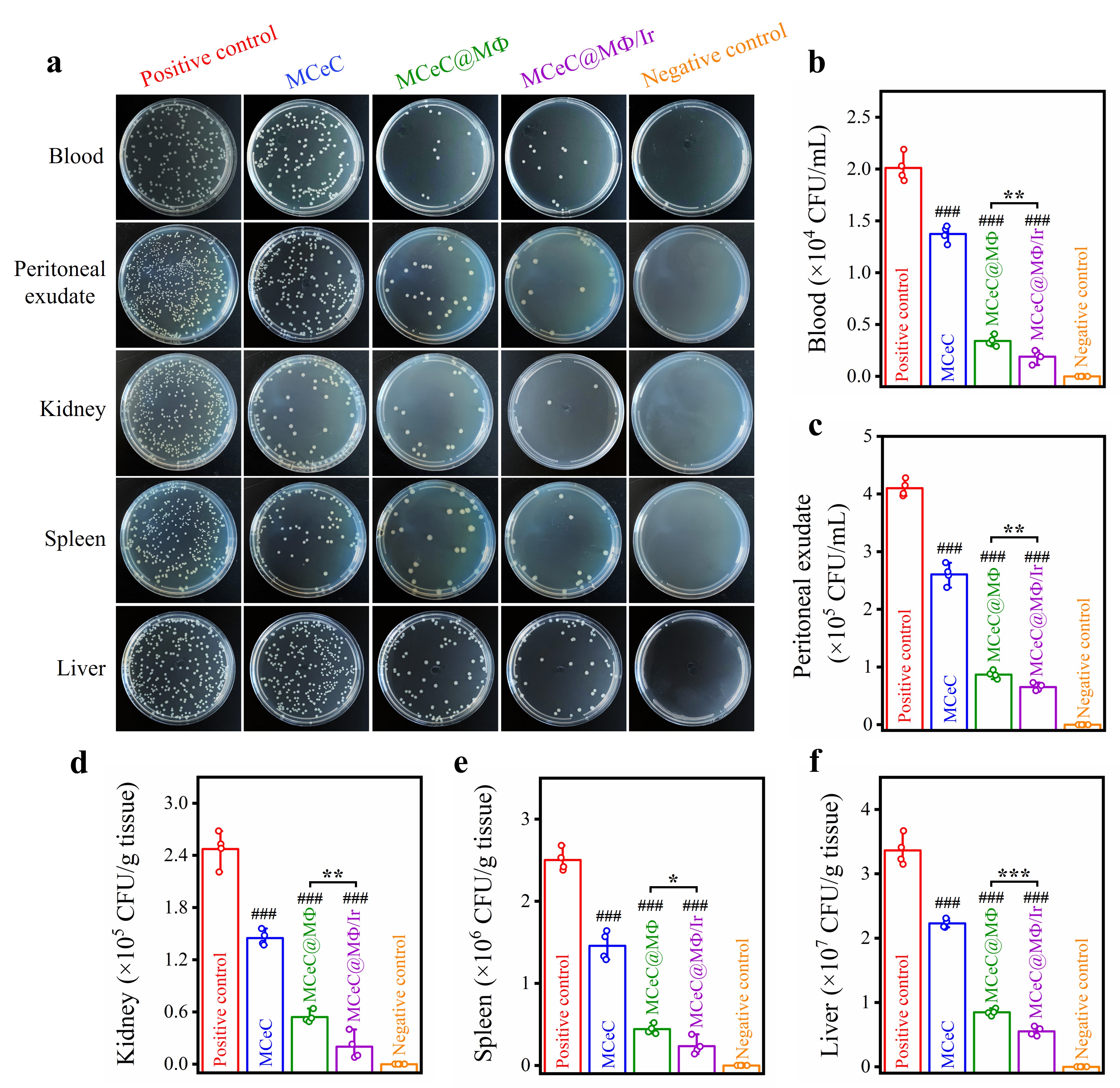


**Figure S21.** Bacterial burden evaluation in septic mice under different treatment conditions. a) Representative photographs of MDR bacterial culture plates taken from blood, peritoneal exudate, and major organs (kidney, spleen, and liver) of septic mice (treated 0.5 h after bacterial inoculation) after 1 day treatment of PBS (positive control), MCeC, MCeC@MΦ, and MCeC@MΦ/Ir, respectively. The group of normal healthy mice was used as the negative control. b-f) Corresponding numbers of bacterial colony shown in (a). The values of bacterial colony number represent the mean of four independent experiments, and the error bars indicate the SD from the mean. # indicates the contrasts between experimental groups and positive control. ^*^*P* < 0.05, ^**^*P* < 0.01, and ^###/***^P < 0.001.


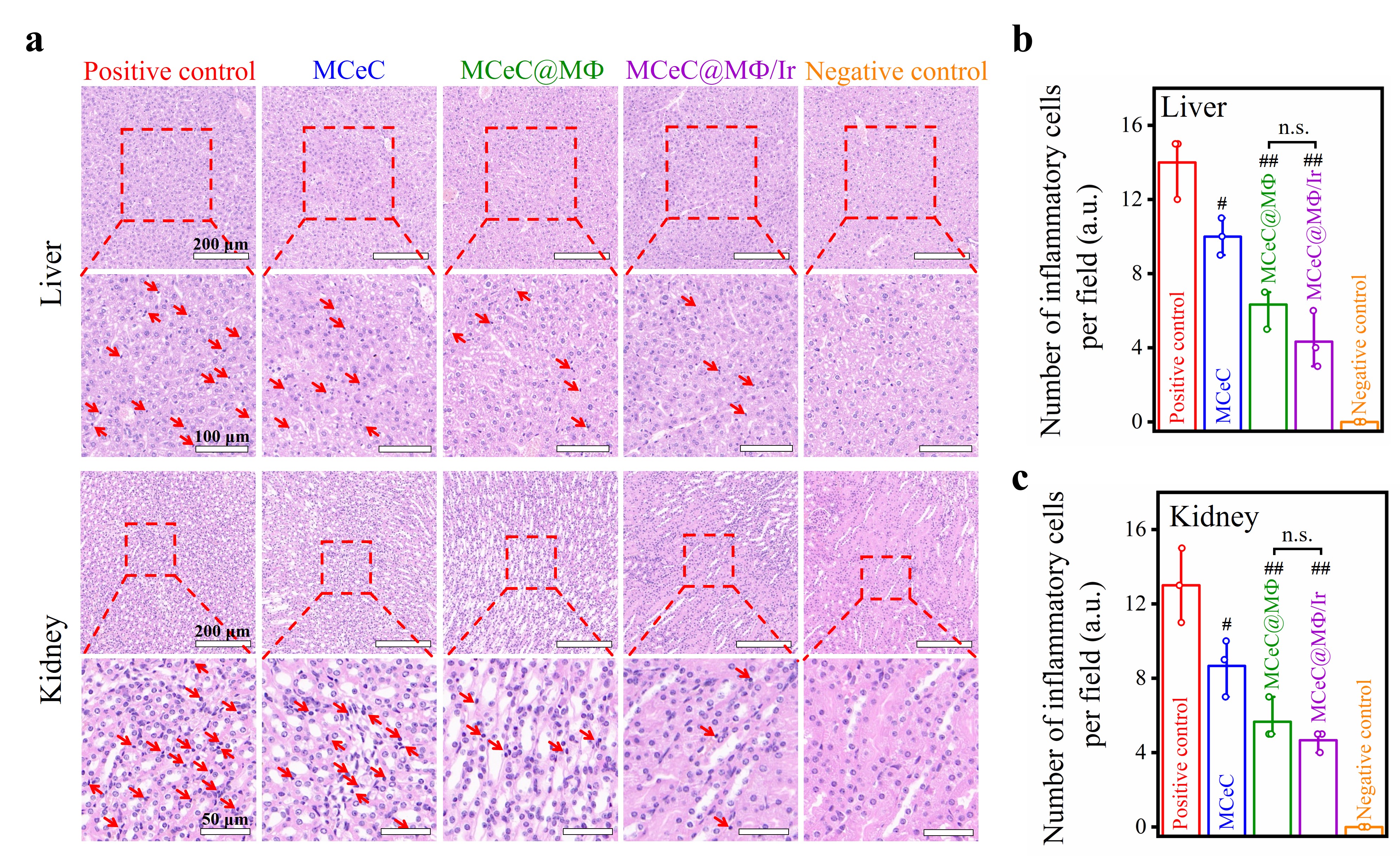


**Figure S22.** Histological analysis of septic mice under different treatment conditions. a) Representative histological (H&E staining) images of the liver and kidney of septic mice (treated 0.5 h after bacterial inoculation) after 1 day treatment of PBS (positive control), MCeC, MCeC@MΦ, and MCeC@MΦ/Ir, respectively. The group of normal healthy mice was used as the negative control. b-c) Corresponding numbers of inflammatory cells shown in (a). The values of inflammatory cell number represent the mean of three independent experiments, and the error bars indicate the SD from the mean. # indicates the contrasts between experimental groups and positive control. ^#^*P* < 0.05, ^##^*P* < 0.01, and ^n.s.^*P* > 0.05.


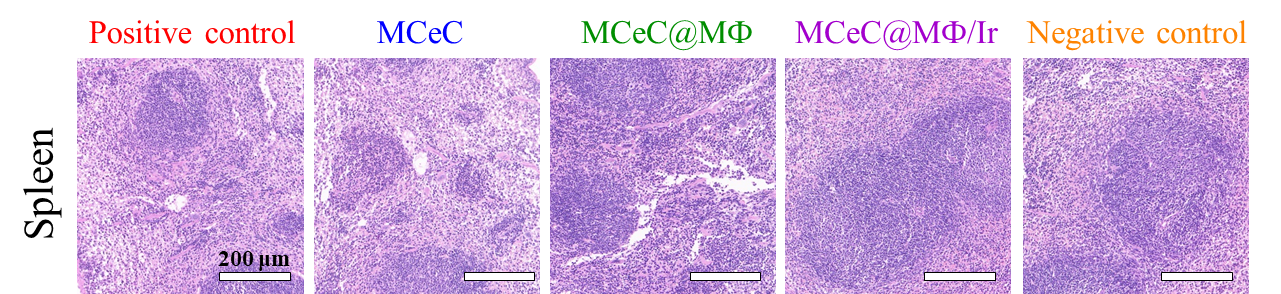


**Figure S23.** Representative histological (H&E staining) images of the spleen of septic mice (treated 0.5 h after bacterial inoculation) after 1 day treatment of PBS (positive control), MCeC, MCeC@MΦ, and MCeC@MΦ/Ir, respectively. The group of normal healthy mice was used as the negative control.





**Figure S24.** The distribution of Ce element in the organs (heart, liver, spleen, lung, and kidney) of healthy mice intraperitoneally injected with MCeC@MΦ at the time point of 24, 48, and 72 h, respectively.


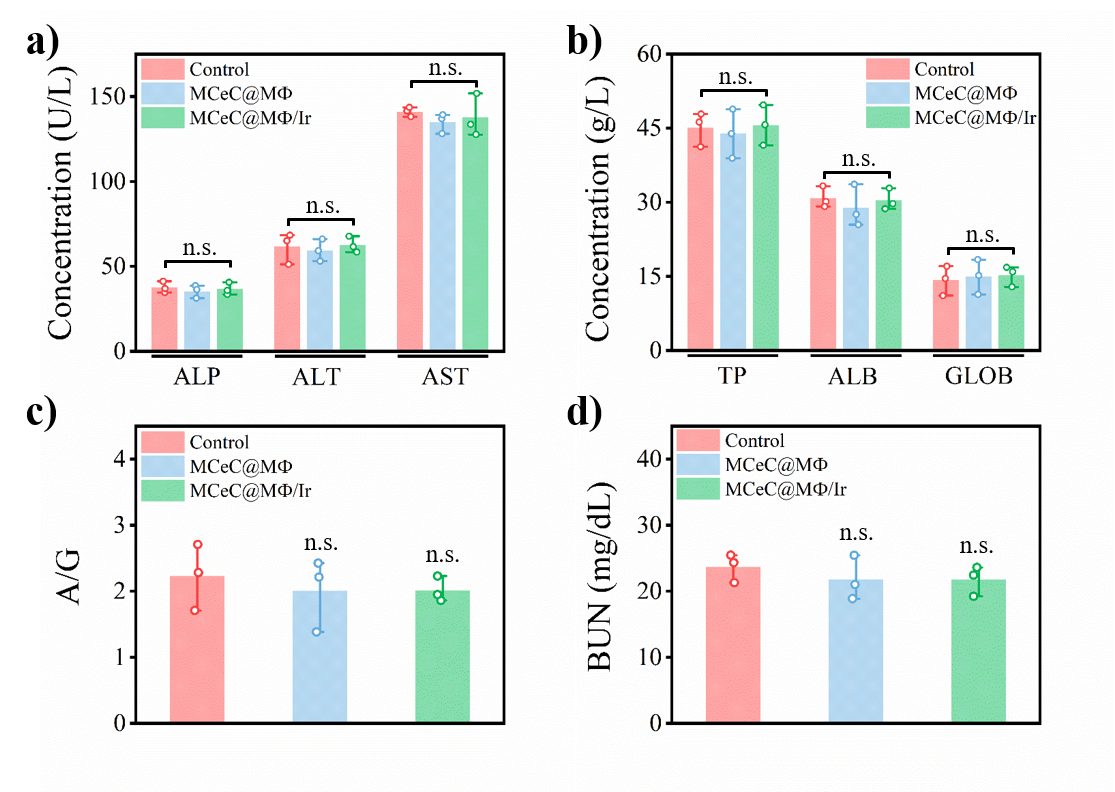


**Figure S25.** Blood biochemistry data of healthy mice on the 5^th^ day of MCeC@MΦ, MCeC@MΦ/Ir, and PBS (control) post-injection, respectively. In these data, TP stands for total protein, GLOB stands for globulin, ALB stands for albumin, A/G stands for the ratio of albumin to globulin, AST stands for aspartate aminotransferase, ALT stands for alanine aminotransferase, ALP stands for alkaline phosphatase, and BUN stands for blood urea nitrogen.


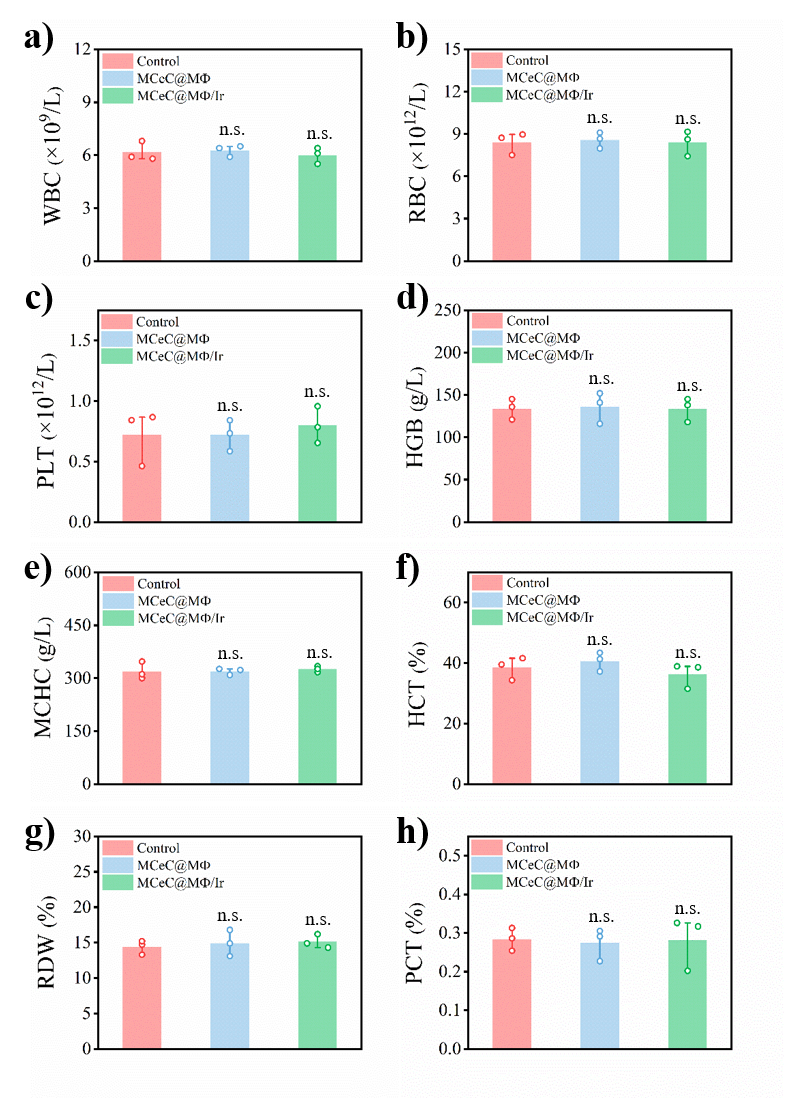


**Figure S26.** Blood routine data of diabetic mice on the 5^th^ day of MCeC@MΦ, MCeC@MΦ/Ir, and PBS (control) post-injection, respectively. In these data, WBC stands for white blood cell, RBC stands for red blood cell, PLT stands for procalcitonin, HGB stands for hemoglobin, MCHC stands for mean erythrocyte hemoglobin concentration, HCT stands for hematocrit, RDW stands for red blood cell distribution width, and PCT stands for procalcitonin.


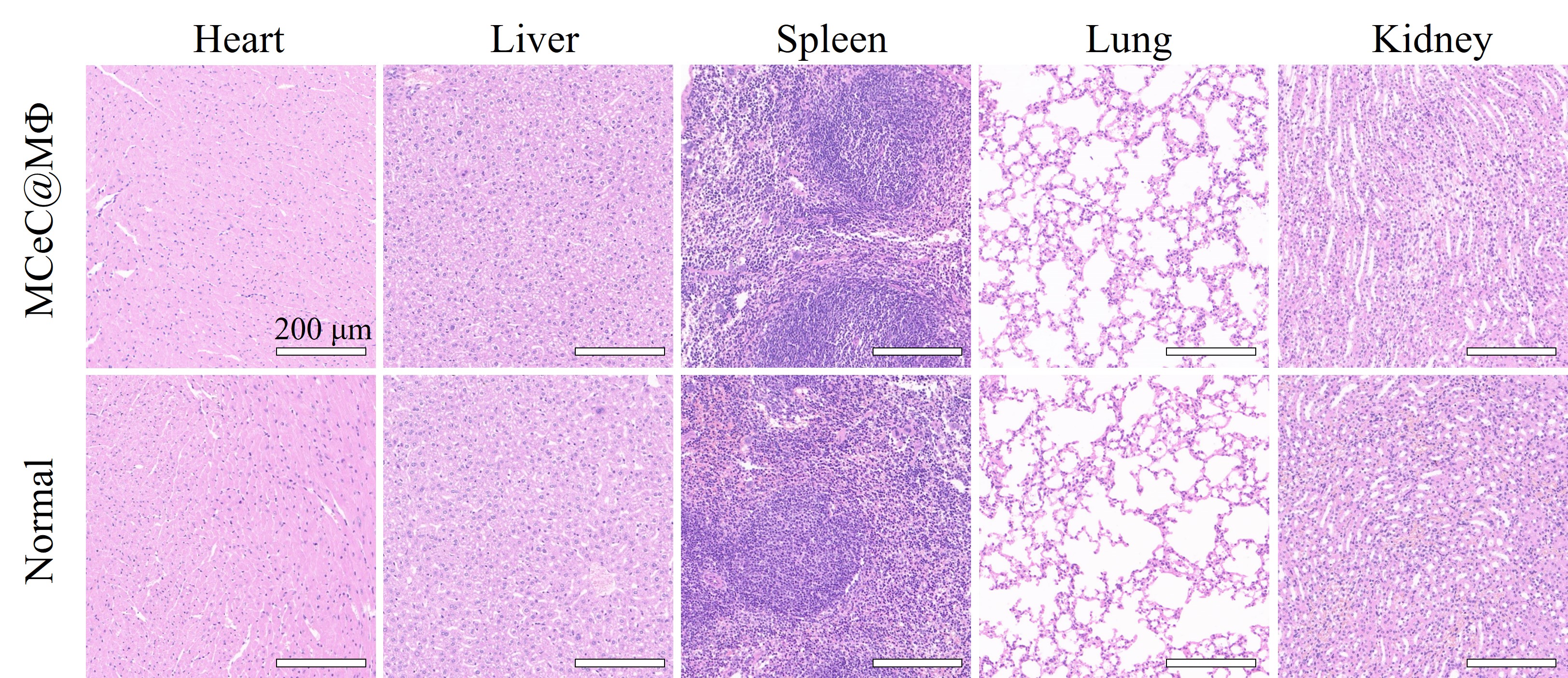


**Figure S27.** Representative H&E staining images of major organs (heart, liver, spleen, lung and kidney) of healthy mice on the 5^th^ day of MCeC@MΦ and PBS (control) post-injection, respectively.
